# Community pharmacy-led diabetes management using continuous glucose monitoring for suboptimally controlled type 2 diabetes: A pilot feasibility study

**DOI:** 10.1371/journal.pone.0350025

**Published:** 2026-05-22

**Authors:** Kyung-In Joung, Ji Won Hwang, So Young Seo, Kyeong Han Back, Yong Geun Jeon, Kwang Joon Kim

**Affiliations:** 1 School of AI Healthcare, CHA University, Pocheon, Republic of Korea; 2 Jeonbuk Pharmaceutical Association, Jeonbuk State, Republic of Korea; 3 College of Pharmacy, Chonnam National University, Gwangju, Republic of Korea; Instituto Nacional de Cardiologia Ignacio Chavez, MEXICO

## Abstract

Many adults with type 2 diabetes mellitus (T2DM) managed without insulin continue to experience suboptimally controlled glycemia. This 12-week, single-arm pilot feasibility study was conducted across 11 community pharmacies in South Korea to evaluate the feasibility and clinical effectiveness of a community pharmacy-led diabetes management model using continuous glucose monitoring (CGM) integrated with digital health platforms. Thirty adults with suboptimally controlled T2DM (HbA1c ≥ 6.5%) on stable oral regimens were enrolled. The intervention combined medication, exercise, and nutrition counseling using CGM (Abbott LibreView®) integrated with the national Personal Health Record system. The primary composite endpoint was defined as achieving all three at week 12: (1) HbA1c ≤ 7.0%, (2) meaningful HbA1c reduction (≥0.5% absolute or ≥10% relative), and (3) time in range (TIR, 70–180 mg/dL) >70%. Results showed that all 30 participants completed the program, with 36.7% achieving the primary composite endpoint. At week 12, HbA1c decreased by 0.70% (95% CI: –1.00 to –0.39; p < 0.001), TIR increased by 5.8 percentage points (p < 0.001), and time above range declined. Time below range remained stable, confirming safety. Improvements appeared from week 3 and were sustained, with longer diabetes duration independently predicting response (adjusted OR=1.24/year, p = 0.040). In conclusion, community pharmacy-led diabetes management using CGM, enabled by digital health integration, produced clinically meaningful and sustained glycemic improvements among adults with T2DM not using insulin. (Trial Registration: KCT0010933).

## Introduction

Type 2 diabetes mellitus (T2DM) poses a substantial and growing global health burden, demanding effective management strategies to mitigate complications and reduce healthcare costs [1–3]. Despite advancements in therapeutic options, a significant proportion of T2DM patients do not consistently achieve individualized glycemic targets, highlighting an ongoing need for more effective and patient-centered interventions [4].

Continuous Glucose Monitoring (CGM) provides detailed, real-time data on glycemic patterns and variability, complementing traditional intermittent glucose measurements [5–7]. International consensus guidelines establish Time in Range (TIR) >70% as a key clinical endpoint correlated with reduced diabetes complications [5,6]. Recent systematic reviews and meta-analyses have demonstrated CGM’s effectiveness in improving glycemic control in various diabetes populations, although results show considerable variability particularly among non-insulin users [8–10]. The largest systematic review (2,783 patients across 26 RCTs) found modest but significant HbA1c reductions with CGM in T2DM, though the clinical impact varied substantially [10]. Notably, the declining cost of CGM sensors in recent years has created new and increasingly cost-effective opportunities for their use in primary healthcare settings, including community pharmacies, making broader adoption of this technology more feasible [8,11].

Concurrently, community pharmacist-led interventions have demonstrated significant effectiveness in diabetes management [12–15]. Systematic reviews show that patient-centered and interdisciplinary approaches yield the most effective outcomes, particularly when they include comprehensive pharmaceutical care components such as medication management, disease education, and lifestyle counseling [12,16].

However, integrating pharmacist expertise with CGM technology in routine care remains a critical challenge. The potential benefits of such a combined service model are particularly unknown for the large population of insulin-naïve patients with suboptimal glycemic control. This group often lacks structured support, even though existing evidence confirms that professional guidance is crucial for maximizing CGM’s effectiveness beyond what technology-only interventions can achieve [17,18].

Unlike insulin-initiated patients who routinely receive intensive glucose monitoring education and structured follow-up, insulin-naive patients typically manage their condition with fewer clinical touchpoints and limited self-monitoring guidance, making them particularly vulnerable to sustained suboptimal glycemic control.

The high accessibility of community pharmacies, combined with the growing availability of integrated digital health platforms, provides a strong rationale for testing a new care model. This pilot feasibility study was designed to assess a novel community pharmacy-led diabetes management service model utilizing CGM technology for this underserved population. The primary aim was to assess the model’s feasibility (e.g., participant retention and data integration), alongside its preliminary clinical effectiveness on glycemic outcomes and patient characteristics associated with treatment response.

## Materials and methods

### Study design and setting

We conducted this pilot feasibility study using a single-arm, before-and-after design. Our primary goal was to evaluate the feasibility and preliminary clinical effectiveness of a community pharmacy-led diabetes management service model that utilizes CGM. The trial ran from June to December 2024 across 11 community pharmacies in Jeonbuk State, Republic of Korea. The study protocol followed the CONSORT extension guidelines for pilot and feasibility trials [19].

The trial protocol was approved by the Institutional Review Board of Chonnam National University (IRB No. 1040198-240423-HR-068-02) and was retrospectively registered with the Clinical Research Information Service (CRIS) which is an online registration system for clinical studies in Korea and one of the primary registries of the World Health Organization (WHO) International Clinical Trials Registry Platform (ICTRP) (No. KCT0010933). All participants provided written informed consent, and the study was conducted in accordance with the Declaration of Helsinki and Good Clinical Practice guidelines [20]. CGM sensors and related supplies were provided at no cost to participants through study funding.

Sample size determination utilized G*Power analysis [21] in conjunction with established pilot study methodology guidelines [22,23]. The target enrollment of 30 participants was selected considering pilot study design principles, the single-arm intervention nature, and practical recruitment capacity within the community pharmacy network.

### Participants

Participants were adults aged 45–65 years with established T2DM, recruited from participating community pharmacies. The pharmacists at these pharmacies volunteered for the study; they were recruited via the Jeonbuk Pharmaceutical Association and subsequently identified eligible patients from their practice.

To be included, participants had to meet several conditions. Clinically, they needed suboptimal glycemic control (HbA1c ≥ 6.5% within the preceding 12 months) and stable treatment (at least two oral hypoglycemic agents for at least one year). They also needed smartphone capability for the CGM app, willingness to complete the 12-week program, and available baseline clinical data. Key exclusion criteria were current or previous insulin use, type 1 diabetes mellitus, treatment with fewer than two oral antidiabetic medications, severe mobility limitations, anticipated skin sensitivity to CGM sensor adhesives, pregnancy or lactation, and inability to provide informed consent or comply with study procedures.

### Community pharmacy-led diabetes management using CGM

The intervention combined CGM technology with pharmacist-led diabetes management utilizing two established platforms: Korean Ministry of Health and Welfare’s Personal Health Record (PHR) application and Abbott’s LibreView^®^ platform with patient consent via MyData system [24]. An optional digital health app (Health&u^®^, TheJOIN Co., Seoul, Korea) was available for dietary monitoring and step tracking as a supplementary support. The PHR application, provided nationwide through Korea’s single-payer National Health Insurance Service, enabled pharmacists to access participants’ comprehensive medication history (past 12 months) and health screening data (past 10 years) with patient consent for baseline assessment. LibreView^®^ platform facilitated real-time CGM data monitoring, automated 2-week glucose reports, and systematic tracking of TIR, glucose statistics, management indicators, and 24-hour glucose profiles [25].

Eleven community pharmacists first completed an 8-session training program. This covered diabetes pathophysiology, CGM technology, and structured counseling protocols for medication management, exercise, and diet. Each pharmacist then managed 1–6 participants (mean 2.7). The standardized counseling was comprehensive: medication management focused on adherence, hypoglycemia, and interactions; exercise counseling involved individualized daily step goals (7,500–10,000); and dietary guidance centered on meal regularity, low-glycemic substitutions, and protein intake based on body weight.

The intervention was structured in three parts over the 12-week study. It began with a 30–60 minute initial consultation (Week 1) covering PHR data review, CGM sensor placement, and device training. Participants then entered a systematic follow-up phase (Weeks 2–12), which involved alternating biweekly 30-minute face-to-face meetings and 10-minute telephone calls. This follow-up was supported by real-time monitoring: pharmacists continuously reviewed LibreView^®^ data and sent personalized texts to guide adherence, exercise, and diet as needed. All participants used FreeStyle Libre^®^ 2 sensors (Abbott Diabetes Care, Alameda, CA, USA) for the study duration, with replacements every 14 days.

### Outcomes and definitions

The primary endpoint was an individualized composite clinical success designed to address the heterogeneity in baseline glycemic status while maintaining clinically meaningful targets. Given that 36.7% of participants had baseline HbA1c ≤ 7.0% (already at guideline target), we implemented composite criteria defined as simultaneous achievement of all three components at 12 weeks: (1) HbA1c ≤ 7.0% based on American Diabetes Association (ADA) recommendations for most nonpregnant adults [26], AND (2) meaningful HbA1c reduction (≥0.5% absolute OR ≥10% relative [27] reduction from baseline), [28,29] AND (3) time in range (TIR) >70% based on International CGM Consensus recommendations [5].

CGM metrics were assessed weekly throughout the 12-week intervention. CGM parameters were calculated according to international consensus guidelines and included: TIR as percentage of time with glucose 70–180 mg/dL, time above range (TAR) as >180 mg/dL, time below range (TBR) as <70 mg/dL, glucose management indicator (GMI), mean glucose, and coefficient of variation.

Clinical laboratory outcomes were measured at three time points: 3 months pre-intervention, baseline, and 12 weeks post-intervention. Primary diabetes-related parameters included HbA1c, fasting blood glucose, and number of oral antidiabetic medications. Additional cardiometabolic parameters included blood pressure, lipid profile, and body mass index.

### Statistical analysis

Descriptive statistics were presented as median (first quartile–third quartile) for continuous variables and frequencies (percentages) for categorical variables. Baseline characteristics were compared between primary composite endpoint achievers and non-achievers using Mann-Whitney U test for continuous variables and Fisher’s exact test for categorical variables.

To analyze the longitudinal changes in CGM metrics, we used generalized estimating equations (GEE), specifying an exchangeable correlation structure [30]. Linear mixed-effects models [31] were employed for clinical outcomes across three time points, with participant ID as a random intercept and time as a fixed effect.

To identify baseline predictors of primary composite endpoint achievement, we performed multivariable logistic regression. We selected five candidate variables based on clinical relevance and univariate results: age, gender, body mass index, diabetes duration, and baseline HbA1c.

Given the small number of outcome events (n = 11 achievers), the multivariable logistic regression model carries a risk of overfitting; results should therefore be interpreted as hypothesis-generating and replicated in larger studies.

We also conducted stratified analyses to examine differential response patterns. Subgroups were defined using 2x2 combinations of significant regression predictors with clinically meaningful cutoffs: age (<60 vs ≥ 60 years), diabetes duration (<10 vs ≥ 10 years), and baseline HbA1c (<7.5% vs ≥ 7.5%). For primary presentation, we selected the combination that showed the greatest differential response.

To assess the robustness of our findings, we performed sensitivity analyses by (1) using alternative composite endpoint definitions with different TIR thresholds (>65%, > 70%, > 75%) (2) conducting a high-risk subgroup analysis (baseline HbA1c ≥ 7.0% and BMI ≥ 25 kg/m²).

We handled missing data differently by type. For the two participants with missing 12-week TIR data, we used conservative imputation: mean substitution for one and last observation carried forward (LOCF) for the other. For missing clinical outcomes, we used full information maximum likelihood estimation within the mixed-effects models.

All analyses were performed using R statistical software (version 4.3.0). Statistical significance was set at p < 0.05.

Given the pilot and exploratory nature of this study, reported p-values should be interpreted as exploratory rather than confirmatory; effect sizes and 95% confidence intervals are emphasized over statistical significance. No adjustment for multiple comparisons was applied to secondary and subgroup analyses given the hypothesis-generating intent of this work.

### Ethics approval and consent to participate

The trial protocol was approved by the Institutional Review Board of Chonnam National University (IRB No. 1040198-240423-HR-068-02) and was retrospectively registered with CRIS which is an online registration system for clinical studies in Korea and one of the primary registries of the WHO ICTRP (No. KCT0010933). Written informed consent was obtained from all participants for study participation.

## Results

### Study enrollment and participant characteristics

**Fig 1:**
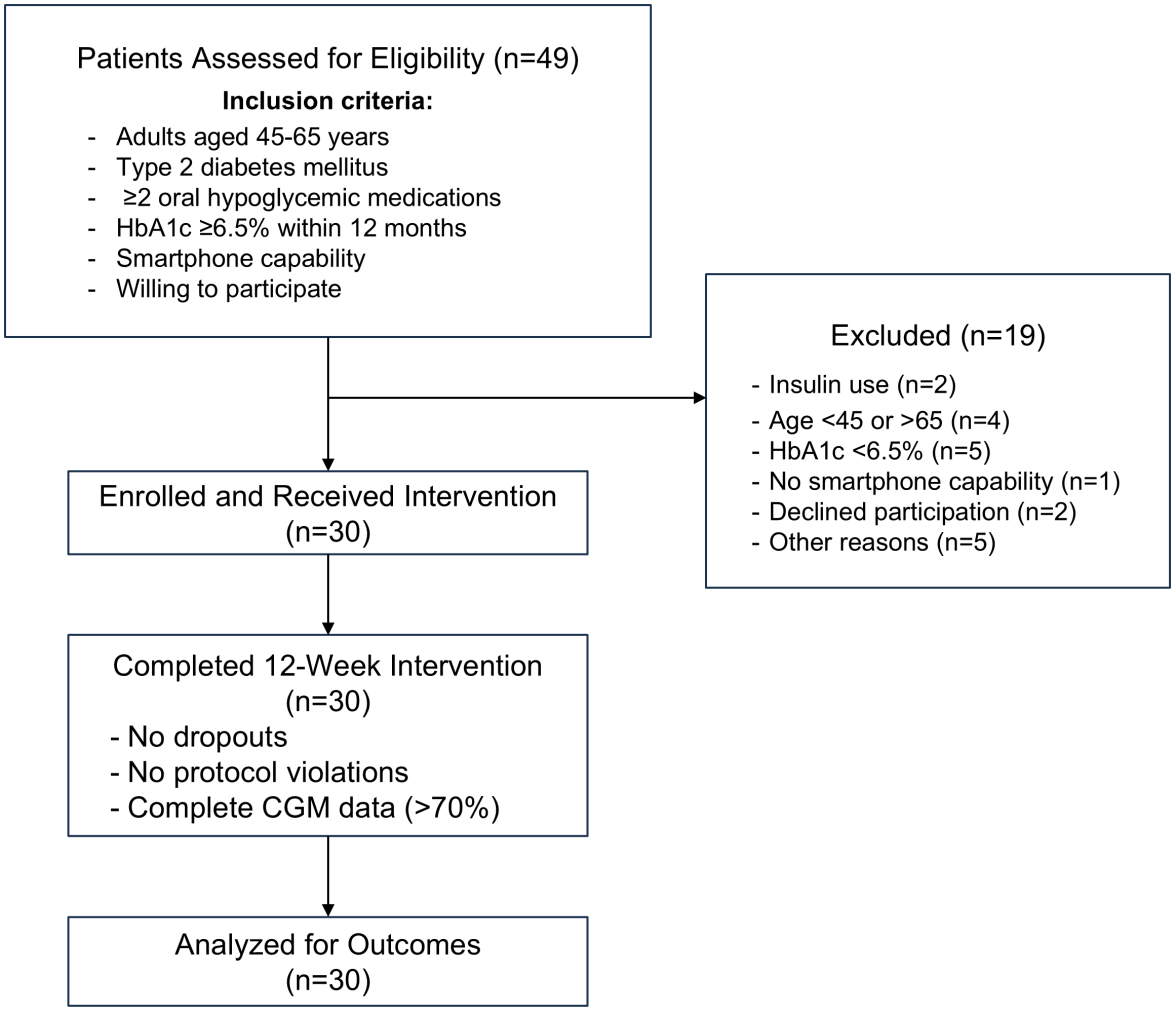
Study flow diagram for the community pharmacy-led diabetes management pilot feasibility study.

Forty-nine adults were assessed for eligibility, with 30 enrolled and completing the 12-week community pharmacy-led diabetes management intervention. Key feasibility outcomes were achieved: 100% participant retention with no protocol violations, and all participants maintained >70% CGM data availability, meeting international consensus guidelines for data quality (Fig 1). These feasibility outcomes confirmed the practicality and acceptability of the model prior to any assessment of clinical effectiveness. Regarding clinical effectiveness outcomes, the following sections present glycemic and cardiometabolic changes observed over the 12-week intervention.

Baseline characteristics demonstrated a real-world diabetes population with heterogeneous glycemic control (Table 1). Median age was 57.00 (53.00–61.00) years with equal gender distribution. Participants had established diabetes with a median duration of 10.00 (5.25–15.00) years and received multiple oral antidiabetic agents [median 3.00 (3.00–4.00) medications]. Median baseline HbA1c was 7.45 (6.90–8.17)%, reflecting suboptimal glycemic control, with median fasting blood glucose of 133.00 (109.00–176.50) mg/dL. Prevalent comorbidities included dyslipidemia (76.7%) and hypertension (43.3%).

**Table 1:** General characteristics of study participants.

Characteristic	Overall (N = 30)	Achievers ^1^ (N = 11)	Non-achievers ² (N = 19)	p-value
Demographics				
Age, years	57.00 (53.00–61.00)	53.00 (52.50–55.00)	60.00 (56.00–61.50)	0.077
Age distribution, n (%)				
45–49 years	2 (6.7)	0 (0)	2 (10.5)	
50–59 years	16 (53.3)	9 (81.8)	7 (36.8)	
60–65 years	12 (40.0)	2 (18.2)	10 (52.6)	
Gender, n (%)				
Male	15 (50.0)	6 (54.5)	9 (47.4)	1.000
Female	15 (50.0)	5 (45.5)	10 (52.6)	
Body mass index, kg/m²	24.26 (21.38–26.48)	25.80 (23.19–28.24)	23.50 (20.94–26.23)	0.197
Medical history				
DM duration, years	10.00 (5.25–15.00)	10.00 (9.00–17.50)	7.00 (4.50–14.50)	0.115
No. of oral antidiabetic medications	3.00 (3.00-4.00)	3.00 (2.50–3.00)	3.00 (3.00–4.00)	0.135
Hypertension, n (%)	13 (43.3)	6 (54.5)	7 (36.8)	0.454
Dyslipidemia, n (%)	23 (76.7)	7 (63.6)	16 (84.2)	0.372
Lifestyle factors				
Current alcohol consumption, n (%)	9 (30.0)	4 (36.4)	5 (26.3)	0.687
Current smoking, n (%)	9 (30.0)	3 (27.3)	6 (31.6)	1.000
Laboratory results				
HbA1c, %	7.45 (6.90–8.17)	7.90 (7.35–8.30)	7.10 (6.80–7.80)	0.058
Fasting blood glucose, mg/dL	133.00 (109.00–176.50)	161.00 (131.00–180.00)	125.00 (107.00–154.50)	0.111
Systolic blood pressure, mmHg	129.00 (112.25–134.75)	128.00 (113.50–132.00)	130.00 (113.00–136.50)	0.667
Total cholesterol, mg/dL	157.00 (129.00–178.00)	152.00 (119.50–194.50)	157.00 (133.00–171.50)	0.966
HDL cholesterol, mg/dL	52.05 (43.00–61.50)	45.00 (42.00–58.00)	54.90 (44.50–61.60)	0.505
LDL cholesterol, mg/dL	64.00 (50.00–89.00)	61.00 (48.30–78.70)	75.00 (52.00–91.00)	0.731
Triglycerides, mg/dL	123.50 (82.00–169.75)	128.00 (105.50–210.00)	115.00 (67.50–156.50)	0.237

Data are presented as median (first quartile–third quartile) for continuous variables and number (percentage) for categorical variables.

Group definitions:

^1^Achievers: Patients who achieved the primary composite endpoint (HbA1c ≤ 7.0% AND meaningful HbA1c reduction (≥0.5% absolute OR ≥10% relative) AND TIR > 70% at 12 weeks).

^2^Non-achievers: Patients who did not achieve the primary composite endpoint.

P-values for between-group comparisons were calculated using Mann-Whitney U test for continuous variables and Fisher’s exact test for categorical variables.

Abbreviations: HbA1c, glycated hemoglobin; BMI, body mass index; DM, diabetes mellitus; HDL, high-density lipoprotein; LDL, low-density lipoprotein.

When comparing primary composite endpoint achievers (n = 11) versus non-achievers (n = 19), baseline characteristics were generally similar with no statistically significant differences in demographics, body mass index, medical history, or laboratory parameters, suggesting that baseline factors alone did not predict treatment success (Table 1).

### Primary composite endpoint achievement and clinical impact

Among the 30 participants, 11 (36.7%, 95% CI: 19.93–56.14) achieved the primary composite endpoint (Table 2). Individual component analysis revealed that 66.7% achieved HbA1c ≤ 7.0%, 56.7% demonstrated meaningful HbA1c reduction, and 66.7% attained TIR > 70%.

**Table 2:** Clinical threshold achievement rates and clinical impact after 12-week pharmacy-led CGM intervention.

Clinical threshold achievement		
Endpoint/ Component	n/N	Achievement rate (95% CI)
**Primary composite** ^1^**endpoint/ component**	11/30	36.7% (19.93–56.14)
**Individual component achievement (N = 30)**		
HbA1c ≤ 7.0% at 12 weeks	20/30	66.7% (48.78–80.77)
Meaningful HbA1c reduction ^2^	17/30	56.7% (39.20–72.62)
≥ 0.50% absolute reduction	17/30	56.7% (39.20–72.62)
≥ 10.00% relative reduction	11/30	36.7% (19.93–56.14)
TIR > 70% at 12 weeks³	20/30	66.7% (48.78–80.77)
**Component failure analysis among non-achievers (n = 19)**		
Failed HbA1c ≤ 7.0% only	1/19	5.3%
Failed meaningful HbA1c reduction only	5/19	26.3%
Failed TIR > 70% only	1/19	5.3%
Failed HbA1c ≤ 7.0% + meaningful HbA1c reduction	3/19	15.8%
Failed HbA1c ≤ 7.0% + TIR > 70%	4/19	21.1%
Failed meaningful HbA1c reduction + TIR > 70%	3/19	15.8%
Failed all three components	2/19	10.5%
Total non-achievers	19/30	
**Baseline HbA1c stratified analysis**		
Baseline HbA1c ≤ 7.00%	2/11	18.2% (5.14–47.70)
Baseline HbA1c 7.01–8.00%	4/10	40.0% (16.82–68.73)
Baseline HbA1c > 8.00%	5/9	55.6% (26.66–81.12)

All proportions estimated using the Clopper-Pearson exact binomial method (95% CI).

^1^Primary composite endpoint: HbA1c ≤ 7.0% AND meaningful HbA1c reduction (≥0.5% absolute OR ≥10% relative) AND TIR > 70%, all met simultaneously at 12 weeks.

^2^Meaningful HbA1c reduction: ≥ 0.50% absolute reduction OR ≥10.00% relative reduction from baseline HbA1c (either criterion sufficient).

^3^Missing 12-week TIR data (n = 2) handled using conservative imputation: mean of weeks 1–8 for one participant (93.9%) and last observation carried forward (LOCF) for the other (100.0%).

Component failure analysis reflects mutually exclusive, exhaustive patterns across the 19 non-achievers.

Abbreviations: CI, confidence interval; HbA1c, glycated hemoglobin; LOCF, last observation carried forward; TIR, time in range.

Baseline HbA1c stratification revealed differential response patterns: participants with baseline HbA1c > 8.0% showed 55.6% achievement rates, those with HbA1c 7.01–8.00% achieved 40.0% success, while those already at target (≤7.0%) demonstrated 18.2% composite endpoint achievement (Table 2).

### Weekly CGM metrics and longitudinal trajectories

The intervention demonstrated progressive improvements across multiple glycemic parameters throughout the 12-week period (Table 3). TIR increased significantly from 72.23 ± 2.78% to 78.07 ± 2.94% (p < 0.001), with meaningful improvements first observed at week 3 and peak performance during weeks 5–6 (80.67–81.40%). This 5.8 percentage point improvement substantially exceeds the clinically meaningful threshold of 5% established by international consensus.

**Table 3:** Weekly changes in CGM metrics during 12-week intervention.

Week	TIR	Mean Glucose	CV	GMI	TAR Level 1	TAR Level 2	Total TAR	TBR Level 1	TBR Level 2	Total TBR
1	72.23 ± 2.78	156.07 ± 4.22	30.94 ± 1.56	7.03 ± 0.09	19.33 ± 1.82	7.23 ± 1.38	26.57 ± 2.80	1.13 ± 0.52	0.07 ± 0.05	1.20 ± 0.52
2	73.97 ± 2.81	152.57 ± 4.10	31.40 ± 1.67	6.96 ± 0.10	17.63 ± 1.84*	6.53 ± 1.22	24.17 ± 2.82	1.53 ± 0.70	0.10 ± 0.07	1.63 ± 0.75
3	76.69 ± 2.74*	146.48 ± 4.58*	30.85 ± 1.56	6.81 ± 0.11*	15.72 ± 1.76**	5.38 ± 1.24	21.10 ± 2.78*	2.07 ± 0.78	0.10 ± 0.07	2.17 ± 0.83
4	78.45 ± 2.68**	142.93 ± 4.45**	29.79 ± 1.56	6.78 ± 0.11**	15.52 ± 1.94*	4.62 ± 1.05	20.14 ± 2.76**	1.69 ± 0.59	0.10 ± 0.07	1.79 ± 0.63
5	80.67 ± 2.44***	141.53 ± 3.54***	29.00 ± 1.45*	6.69 ± 0.08***	14.57 ± 1.77**	3.20 ± 0.69**	17.77 ± 2.32***	1.50 ± 0.66	0.10 ± 0.07	1.60 ± 0.72
6	81.40 ± 2.33***	140.37 ± 3.44***	29.42 ± 1.48	6.67 ± 0.08***	13.73 ± 1.61***	3.50 ± 0.80**	17.23 ± 2.26***	1.63 ± 0.71	0.07 ± 0.05	1.70 ± 0.74
7	80.27 ± 2.50***	141.50 ± 3.83**	30.24 ± 1.75	6.70 ± 0.09***	13.87 ± 1.65***	4.07 ± 0.98*	17.93 ± 2.40***	1.87 ± 0.67	0.20 ± 0.17	2.07 ± 0.80
8	81.00 ± 2.51***	141.23 ± 3.64***	29.87 ± 1.80	6.69 ± 0.09***	13.43 ± 1.62***	3.80 ± 0.86**	17.23 ± 2.31***	1.77 ± 0.71	0.23 ± 0.20	2.00 ± 0.86
9	80.69 ± 2.84***	140.93 ± 3.93***	29.37 ± 1.77	6.67 ± 0.09***	13.93 ± 1.90***	3.66 ± 0.92*	17.59 ± 2.65***	1.59 ± 0.63	0.14 ± 0.14	1.72 ± 0.72
10	80.72 ± 2.78***	140.66 ± 3.66***	28.65 ± 1.55**	6.69 ± 0.08***	14.45 ± 2.01***	3.38 ± 0.84**	17.83 ± 2.67***	1.38 ± 0.39	0.07 ± 0.05	1.45 ± 0.42
11	81.00 ± 2.63***	142.31 ± 3.70***	28.58 ± 1.51**	6.67 ± 0.09***	14.14 ± 1.89***	3.17 ± 0.87**	17.31 ± 2.60***	1.59 ± 0.47	0.10 ± 0.06	1.69 ± 0.50
12	78.07 ± 2.94**	145.29 ± 4.22**	29.27 ± 1.58*	6.72 ± 0.09***	16.07 ± 2.14*	4.46 ± 1.05	20.54 ± 2.96*	1.32 ± 0.48	0.07 ± 0.05	1.39 ± 0.50
Linear Trend *p*	<0.001***	0.004**	0.004**	<0.001***	0.003**	0.006**	<0.001***	0.830	0.824	0.866

Data are presented as mean ± standard error.

* *p* < 0.05, ** *p* < 0.01, *** *p* < 0.001.

Asterisks in weeks 2–12 indicate significant difference vs. baseline (week 1) using GEE analysis.

Linear Trend P shows p-value for overall linear trend across 12 weeks with significance levels.

Missing data: Two participants had incomplete CGM follow-up (one missing weeks 9–12, one missing week 12). GEE analysis included all available observations.

Abbreviations: CGM, continuous glucose monitoring; GMI, glucose management indicator, TIR, time in range; TAR, time above range; TBR, time below range; CV, coefficient of variation; GEE, generalized estimating equations.

Complementary improvements included significant reductions in mean glucose (156.07 to 145.29 mg/dL, p = 0.004), glucose management indicator (7.03% to 6.72%, p < 0.001), and glycemic variability as measured by coefficient of variation (30.94% to 29.27%, p = 0.004). Time above range decreased substantially from 26.57 ± 2.80% to 20.54 ± 2.96% (p < 0.001), driven by improvements in both Level 1 and Level 2 hyperglycemia (both p ≤ 0.006). Time below range remained stable and low throughout the study (1.20% to 1.39%, p = 0.866). The temporal patterns revealed initial improvement within 3 weeks, optimal performance during weeks 4–8, and sustained benefits through study completion.

**Fig 2:**
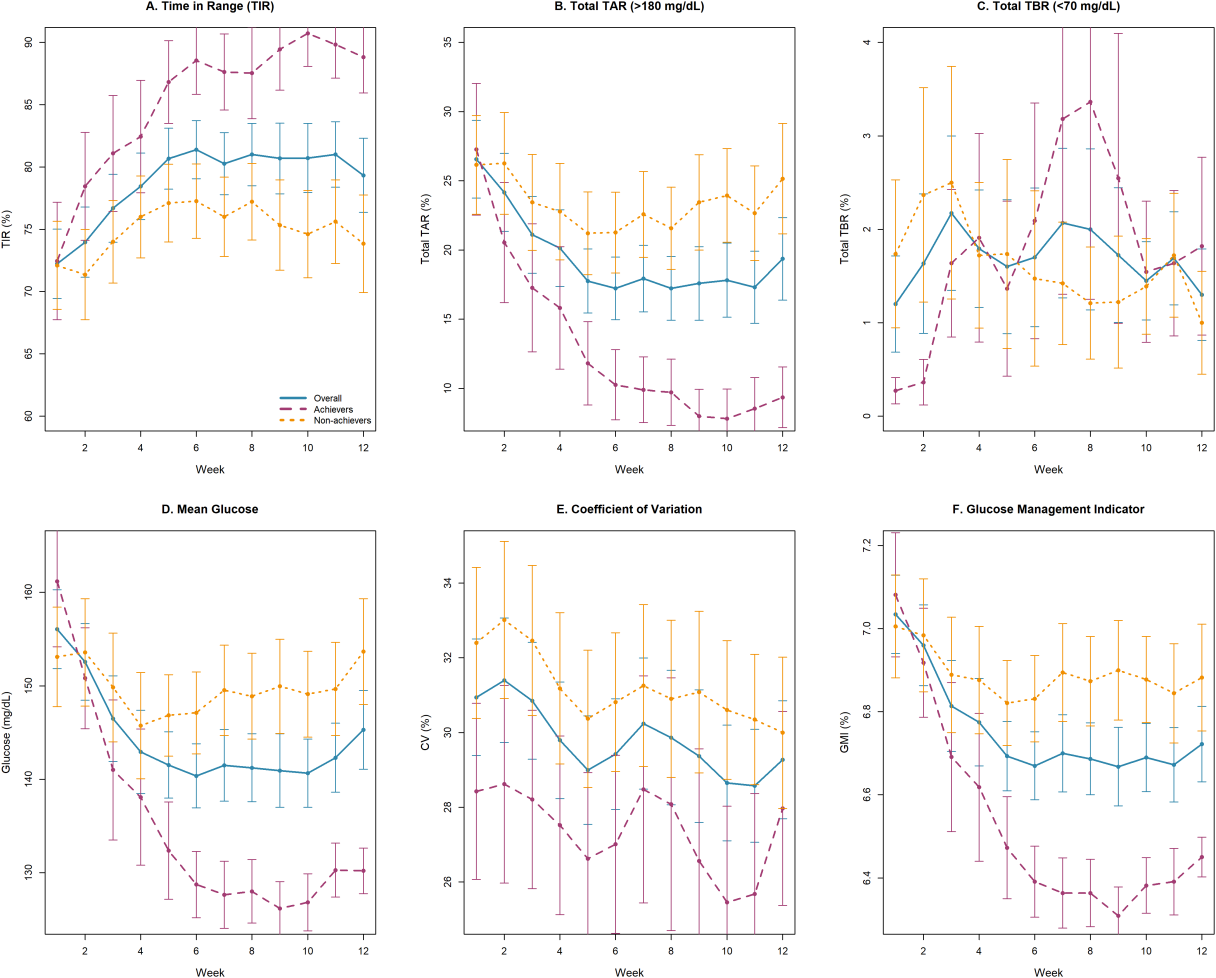
Longitudinal changes in continuous glucose monitoring metrics over 12 weeks: comparison between primary composite endpoint achievers and non-achievers. Data presented as mean ± standard error analyzed using generalized estimating equations (GEE). Panels show: **(A)** Time in Range (TIR, 70-180 mg/dL), **(B)** Total Time Above Range (TAR, > 180 mg/dL), **(C)** Total Time Below Range (TBR, < 70 mg/dL), **(D)** Mean glucose, **(E)** Coefficient of variation, and **(F)** Glucose Management Indicator. Primary composite endpoint achievers (purple dashed line), non-achievers (orange dotted line). Missing data handled through GEE methodology. Abbreviations: CGM, continuous glucose monitoring; GEE, generalized estimating equations.

Fig 2 illustrates the longitudinal CGM trajectories for all 30 participants over the 12-week intervention. The entire cohort showed consistent improvements in key glycemic parameters, with time below range (TBR) remaining consistently low and stable. This overall temporal pattern included a progressive enhancement in TIR that began at week 3, peaked during weeks 5–6, and was sustained through the study’s completion. The subgroup analysis revealed distinct response trajectories. Achievers demonstrated consistently superior improvements, which contrasted sharply with the more modest changes observed in non-achievers.

### Clinical outcomes across study periods

Linear mixed-effects analysis across three time points demonstrated significant glycemic improvement following the intervention (Table 4). HbA1c remained unchanged during the pre-intervention period (7.61 ± 0.94% to 7.61 ± 0.87%, p = 0.824) but decreased significantly from baseline to 12 weeks (7.61 ± 0.87% to 6.91 ± 0.63%, p < 0.001), representing a 0.70% absolute reduction.

**Table 4:** Changes in clinical outcomes across three time points using Linear Mixed-Effect model (Pre-intervention, Baseline, and 12 weeks).

Variable	N	Pre-3mo	Baseline	Post-12w	Δ(1–2)^1^, 95% CI	p-value (1–2)^1^	Δ(2–3)^2^, 95% CI	p-value (2–3)^2^	*P-*value (overall)
HbA1c (%)	30	7.61 ± 0.94	7.61 ± 0.87	6.91 ± 0.63	−0.03 (−0.31, 0.25)	0.824	−0.70 (−1.00, −0.39)	<0.001	<0.001
FBG (mg/dL)	30	169.95 ± 70.22	150.93 ± 63.01	134.24 ± 35.01	−17 (−58.53, 24.53)	0.404	−17.79 (−41.96, 6.37)	0.143	0.143
Systolic BP (mmHg)	30	125.11 ± 17.26	125.83 ± 14.82	123.2 ± 12.95	4 (−4.96, 12.96)	0.334	−2.63 (−6.43, 1.16)	0.167	0.167
Diastolic BP (mmHg)	30	74.89 ± 11.11	75.97 ± 10.48	75.27 ± 9.3	1.56 (−11.98, 15.09)	0.798	−0.70 (−3.65, 2.25)	0.631	0.631
Total cholesterol (mg/dL)	30	168.25 ± 39.19	156.13 ± 40.53	148.83 ± 38.49	−11.5 (−29.44, 6.44)	0.198	−7.30 (−14.39, −0.21)	0.044	0.044
HDL (mg/dL)	30	58.37 ± 13.68	53.34 ± 13.92	54.04 ± 12.41	−1.92 (−6.11, 2.27)	0.352	0.70 (−2.56, 3.96)	0.662	0.352
LDL (mg/dL)	29	81.73 ± 35.96	72.07 ± 32.01	70.35 ± 33.53	−7.73 (−26.03, 10.58)	0.390	−4.10 (−11.45, 3.25)	0.263	0.263
TG (mg/dL)	30	164.74 ± 113.71	156.47 ± 149.3	136.67 ± 106.04	−30.87 (−82, 20.26)	0.224	−19.80 (−59.37, 19.77)	0.315	0.224
BMI (kg/m2)	30	N/A	25.41 ± 5.18	24.83 ± 4.92	N/A	N/A	−0.58 (−1.01, −0.14)	0.012	0.012
No. of oral antidiabetic medications	30	3.20 ± 0.85	3.20 ± 0.81	3.07 ± 0.87	0.00 (−0.10, 0.10)	1.000	−0.13 (−0.32, 0.06)	0.161	0.143

Data are presented as estimated marginal means ± standard error derived from linear mixed-effects models.

BMI was measured only at baseline and 12-week follow-up (pre-intervention measurement not available); overall p-value represents baseline vs 12-week comparison.

Statistical analysis was conducted using linear mixed-effects models with participant ID as a random intercept effect and time point as a fixed effect. This analytical approach accommodates missing data through maximum likelihood estimation, allowing inclusion of all participants regardless of incomplete observations at specific time points. The compound symmetry covariance structure was specified for the repeated measures.

Pairwise comparisons between time points were performed using estimated marginal means with Bonferroni adjustment for multiple comparisons: ¹ Δ(1–2): Pre-intervention (3 months prior) vs Baseline (Week 0) ² Δ(2–3): Baseline (Week 0) vs 12 weeks after intervention.

P-value (overall) represents the omnibus F-test for the overall time effect across all three measurement points.

Missing data were handled under the missing at random (MAR) assumption using full information maximum likelihood estimation. Model assumptions including normality of residuals and homoscedasticity were verified through diagnostic plots.

Abbreviations: HbA1c, glycated hemoglobin; FBG, fasting blood glucose; BP, blood pressure; HDL, high-density lipoprotein; LDL, low-density lipoprotein; TG, triglyceride; CI, confidence interval; BMI, body mass index; DM, diabetes mellitus.

The number of oral antidiabetic medications showed a numerical decrease from 3.20 ± 0.81 to 3.07 ± 0.87, although this change did not reach statistical significance (p = 0.161). Among secondary clinical outcomes, BMI decreased significantly (−0.58 kg/m², 95% CI: −1.01 to −0.14, p = 0.012) and total cholesterol showed modest reduction (−7.3 mg/dL, 95% CI: −14.39 to −0.21, p = 0.044). Fasting blood glucose, blood pressure parameters, and other lipid measures demonstrated no significant changes.

### Predictive modeling and response patterns

Comparison of achievers versus non-achievers revealed significant differences in treatment response (Table 5). Achievers demonstrated significantly greater improvements in HbA1c absolute reduction [−1.10 (−1.85 to −0.90)% vs −0.20 (−0.50 to 0.00)%, p < 0.001], TIR improvement [17.00 (11.00 to 24.25)% vs 5.00 (−3.25 to 6.75)%, p = 0.004], mean glucose reduction [−37.50 (−45.00 to −18.00) vs −4.00 (−13.25 to 3.50) mg/dL, p = 0.004], and BMI reduction [−1.32 (−2.21 to −0.37) vs 0.00 (−0.18 to 0.25) kg/m², p < 0.001].

**Table 5:** Clinical and CGM outcomes by primary composite endpoint achievement category: changes from baseline to 12 Weeks).

Variable	Achievers (n = 11)	Non-achievers (n = 19)	p-value
HbA1c change (Absolute), %	−1.10 (−1.85 to −0.90)	−0.20 (−0.50 to 0.00)	<0.001
HbA1c change (Relative), %	−14.29 (−22.31 to −12.51)	−2.99 (−6.50 to 0.00)	<0.001
Fasting blood glucose, mg/dL	−24.00 (−35.50 to −12.00)	2.00 (−7.50 to 11.25)	0.033
TIR (%) change	17.00 (11.00 to 24.25)	5.00 (−3.25 to 6.75)	0.004
Mean glucose (mg/dL) change	−37.50 (−45.00 to −18.00)	−4.00 (−13.25 to 3.50)	0.004
Total TAR (%) change	−17.00 (−27.00 to −12.00)	−4.00 (−6.50 to 2.50)	0.003
Total TBR (%) change	0.00 (0.00 to 1.50)	0.00 (−0.50 to 0.00)	0.140
Systolic blood pressure, mmHg	0.00 (−7.00 to 8.50)	0.00 (−12.00 to 5.00)	0.477
Total cholesterol, mg/dL	−8.00 (−25.50 to −4.00)	−1.00 (−11.00 to 1.50)	0.174
BMI (kg/m²) change	−1.32 (−2.21 to −0.37)	0.00 (−0.18 to 0.25)	<0.001
No. of oral antidiabetic medications	0.00 (0.00 to 0.00)	0.00 (0.00 to 0.00)	0.388

Data are presented as median (Q1–Q3). Between-group comparisons were performed using Mann-Whitney U test.

Primary Composite Endpoint: Achievers achieved HbA1c ≤7.0% at 12 weeks AND Meaningful HbA1c reduction (≥0.5% absolute OR ≥10% relative) AND TIR >70% at week 12. Non-Achievers did not meet all criteria.

Change calculations: All changes were calculated as 12-week value minus baseline value. Negative values indicate improvement for HbA1c, TAR, clinical measures, and medication count. Positive values indicate improvement for TIR.

CGM Definitions: TIR = 70-180 mg/dL; TAR = >180 mg/dL; TBR = <70 mg/dL.

Mean glucose change analysis based on N=28 due to incomplete CGM data in 2 participants. Statistics: All tests two-sided (α = 0.05). No multiple comparison adjustment applied in this study.

Abbreviations: CGM, continuous glucose monitoring; HbA1c, glycated hemoglobin; TIR, time in range; TAR, time above range; TBR, time below range; FBG, fasting blood glucose; BMI, body mass index.

Multivariable logistic regression analysis identified diabetes duration as the only significant independent predictor of primary composite endpoint achievement (adjusted OR=1.24 per year, 95% CI: 1.01–1.53, p = 0.040) (Table 6). Age showed a borderline significant association (adjusted OR=0.81, 95% CI: 0.64–1.02, p = 0.072), while gender, BMI, and baseline HbA1c were not significantly associated with treatment response.

**Table 6:** Predictors of primary composite endpoint achievement after 12-week CGM-Based intervention.

Variable	Achievers (n = 11)	Non-achievers (n = 19)	P-value¹	Crude OR (95% CI)²	P-value²	Adjusted OR (95% CI)³	P-value³
Age (years)	54.73 ± 4.36	58.05 ± 5.3	0.075	0.87 (0.74–1.02)	0.096	0.81 (0.64–1.02)	0.072
Gender (Male), n (%)	5 (45.45)	10 (52.63)	1	1.33 (0.3–5.92)	0.705	1.87 (0.25–13.92)	0.539
BMI (kg/m²)	26.84 ± 5.65	24.58 ± 4.85	0.28	1.09 (0.94–1.27)	0.258	1.07 (0.88–1.31)	0.491
DM Duration (years)	12.91 ± 6.71	9.11 ± 5.56	0.129	1.11 (0.98–1.27)	0.111	1.24 (1.01–1.53)	0.040
Baseline HbA1c (%)	7.89 ± 0.75	7.45 ± 0.91	0.165	1.83 (0.75–4.49)	0.187	1.37 (0.47–3.99)	0.567

Primary composite endpoint: Achievers achieved HbA1c ≤7.0% at 12 weeks AND meaningful HbA1c reduction (≥0.5% absolute OR ≥10% relative) AND TIR >70% at week 12. Non-achievers did not meet all criteria.

^1^P-values for between-group comparisons using independent t-test for continuous variables and Fisher’s exact test for categorical variables.

^2^Crude odds ratios (OR) and 95% confidence intervals (CI) from univariate logistic regression analysis. OR represents the odds of achieving high response per 1-unit increase for continuous variables, or male vs. female for gender.

^3^Adjusted odds ratios and 95% confidence intervals from multivariable logistic regression including all five predictor variables simultaneously.

^4^Statistically significant at α = 0.05.

Abbreviations: BMI, body mass index; CI, confidence interval; DM, diabetes mellitus; HbA1c, glycated hemoglobin; OR, odds ratio.

Model Performance: The multivariable logistic regression model included 30 patients with complete data. AIC (Akaike Information Criterion) = 30.83, McFadden’s Pseudo R² = 0.303, indicating moderate model fit.

### Stratified analysis and response heterogeneity

**Fig 3:**
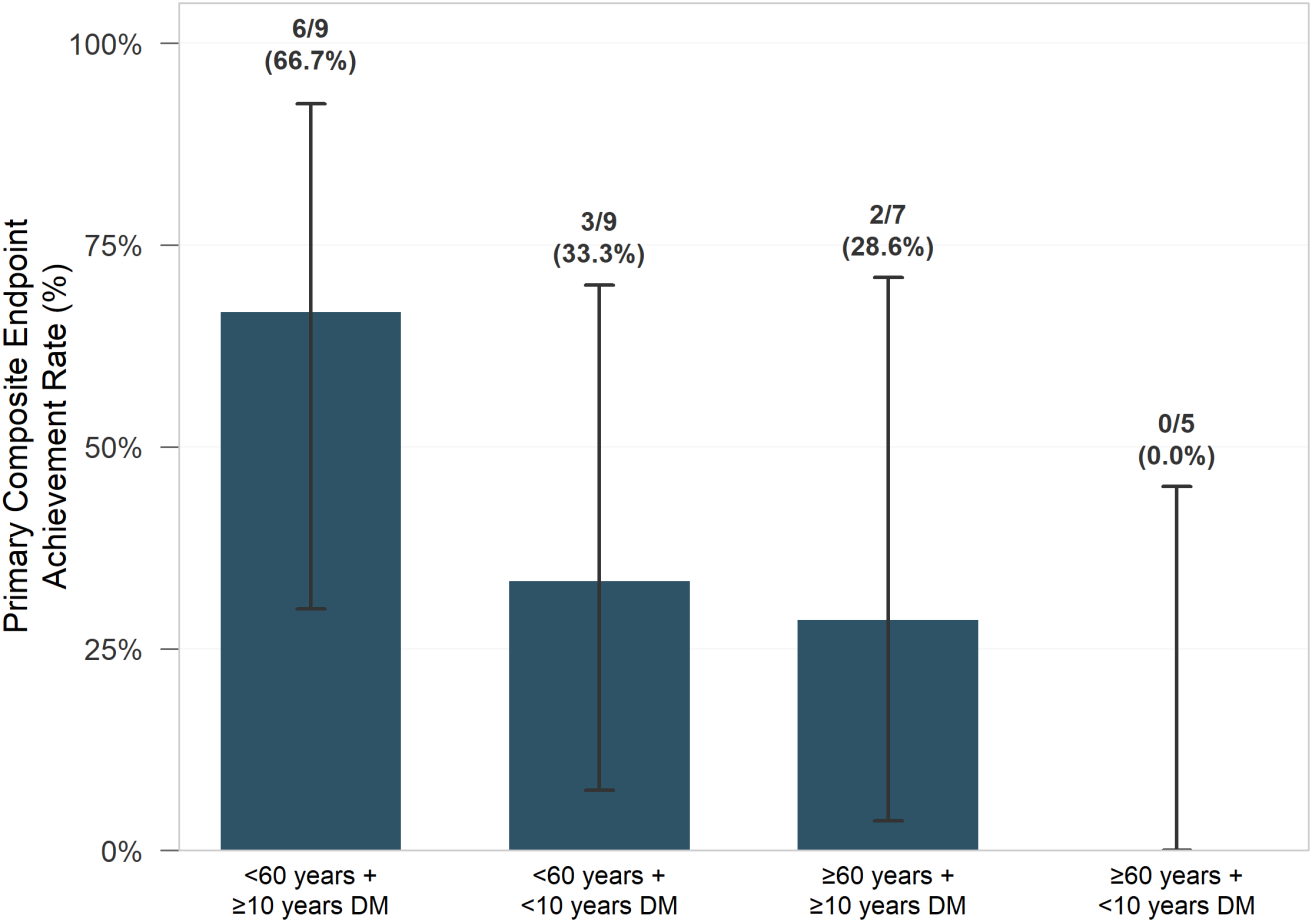
Primary composite endpoint achievement rates stratified by age and diabetes duration: differential response patterns in pharmacist-supported CGM intervention. Primary composite endpoint defined as achieving HbA1c ≤ 7.0% AND meaningful HbA1c reduction (≥0.5% absolute OR ≥10% relative) AND TIR > 70% at 12 weeks. Values above bars indicate number of events/total number (achievement rate). Error bars represent 95% confidence intervals calculated using Clopper-Pearson method. Abbreviations: CGM, continuous glucose monitoring; DM, diabetes mellitus; HbA1c, glycated hemoglobin; TIR, time in range.

Stratified analysis revealed substantial heterogeneity in treatment response, with achievement rates ranging from 0% to 66.7% across different patient subgroups (Table 7, Fig 3). The age × diabetes duration combination demonstrated the greatest differential response among tested stratification schemes. Younger patients with longer diabetes duration (≥10 years) showed the highest achievement rates (6/9 patients, 66.7%), while older patients with shorter diabetes duration (<10 years) showed no treatment response (0/5 patients, 0%).

**Table 7:** Stratified analysis of primary composite endpoint achievement by baseline predictors.

Stratification variables	Group combination	N	Events	Rate (95% CI)
Age x DM duration*	<60 years + ≥10 years DM	9	6	66.67% (35.42–87.94)
	<60 years + < 10 years DM	9	3	33.33% (12.06–64.58)
	≥60 years + ≥10 years DM	7	2	28.57% (8.22–64.11)
	≥60 years + < 10 years DM	5	0	0.00% (0.00–43.45)
Age x Baseline HbA1c	<60 years + Baseline HbA1c ≥ 7.5%	11	7	63.64% (35.38–84.83)
	<60 years + Baseline HbA1c < 7.5%	7	2	28.57% (8.22–64.11)
	≥60 years + Baseline HbA1c ≥ 7.5%	4	1	25.00% (4.56–69.94)
	≥60 years + Baseline HbA1c < 7.5%	8	1	12.50% (2.24–47.09)
DM Duration x Baseline HbA1c	≥10 years DM + Baseline HbA1c ≥ 7.5%	9	5	55.56% (26.67–81.12)
	<10 years DM + Baseline HbA1c ≥ 7.5%	6	3	50.00% (18.76–81.24)
	≥10 years DM + Baseline HbA1c < 7.5%	7	3	42.86% (15.82–74.95)
	<10 years DM + Baseline HbA1c < 7.5%	8	0	0.00% (0.00–32.44)

Selected combination based on maximum differential response pattern.

Variable Definitions:

Age groups: < 60 years vs ≥ 60 years

DM Duration: < 10 years vs ≥ 10 years

Baseline HbA1c: < 7.5% vs ≥ 7.5%

Primary composite endpoint: HbA1c ≤ 7.0% AND meaningful HbA1c reduction (≥0.5% absolute OR ≥10% relative) AND TIR > 70% at 12 weeks.

Variable selection based on multivariable logistic regression results (Table 6): DM duration (p = 0.040), age (p = 0.072), baseline HbA1c (p = 0.187).

Range represents the absolute difference between highest and lowest achievement rates within each stratification scheme.

Abbreviations: CI, confidence interval; DM, diabetes mellitus; HbA1c, glycated hemoglobin; TIR, time in range.

Additional stratification schemes showed smaller differentials: age × baseline HbA1c demonstrated a 51.1% range (63.6% vs 12.5%), while diabetes duration × baseline HbA1c showed a 55.6% range (55.6% vs 0%).

Within the younger age stratum (<60 years), achievers demonstrated significantly greater improvements in HbA1c [−1.30 (−2.00 to −1.00)% vs −0.20 (−0.50 to 0.00)%, p < 0.001], and TIR [+17.00 (11.00 to 19.00)%p vs −1.00 (−4.00 to 7.00)%p, p = 0.008] compared with non-achievers (S2 Table).

These exploratory findings suggest that younger age combined with longer diabetes duration may identify patients more likely to respond to CGM-guided care, consistent with diabetes duration being the only significant independent predictor in multivariable analysis.

### Sensitivity analysis

Sensitivity analyses confirmed the robustness of primary findings across different analytical approaches. When varying the TIR threshold from >65% to >75%, achievement rates ranged from 33.3% to 40.0%, with our primary threshold of >70% yielding an intermediate rate of 36.7%. Alternative HbA1c reduction criteria showed achievement rates of 36.7% for absolute reduction only and 30.0% for relative reduction only (S1 Table).

## Discussion

Our pilot feasibility study showed that the community pharmacy-led, CGM-guided diabetes management model is both feasible and clinically effective. We observed significant glycemic improvements: 36.7% of participants achieved the primary composite endpoint (95% CI: 19.93–56.14), HbA1c decreased by 0.70% (p < 0.001), and TIR increased by 5.8 percentage points (p < 0.001). These TIR improvements began at week 3 and were sustained. The intervention also provided broader cardiometabolic benefits, such as significant reductions in BMI (p = 0.012) and total cholesterol (p = 0.044).

Evaluating treatment benefits in diabetes studies is challenging when participants have heterogeneous baseline glycemic control. In our study, 36.7% of participants already had an HbA1c ≥ 7.0% at baseline, meaning traditional uniform targets would have failed to capture intervention benefits for them. To address this, our individualized composite endpoint was grounded in three established clinical guidelines: (1) HbA1c ≥ 7.0% (ADA recommendations) [26], (2) meaningful HbA1c reduction (≥0.5% absolute or ≥10% relative) [27–29], and (3) TIR > 70% (International CGM Consensus) [5]. This evidence-based design allowed for a more equitable evaluation across all participants and addressed a key limitation of studies that use uniform targets.

Sensitivity analyses confirmed the robustness of our composite endpoint (S1 Table). We tested several alternative definitions: varying the TIR thresholds (from >65% to >75%), using different HbA1c reduction criteria (absolute-only or relative-only), and changing the TIR measurement timing (4-week average). All variants produced highly consistent achievement rates, ranging from 30.0% to 40.0%. This narrow range supports our primary finding (36.7%) and shows the composite endpoint is a balanced measure.

Regarding the clinical outcomes, the glycemic improvements observed in this study align closely with recent evidence examining CGM use in non-insulin-treated type 2 diabetes populations. Our HbA1c reduction of 0.70% is consistent with randomized controlled trials and real-world studies across diverse settings. Recent trials demonstrate variable but consistent CGM benefits. Martens et al. (2021) showed a 0.4% net HbA1c benefit with higher TIR (59% vs 43%, p < 0.001) in basal insulin-treated patients [32]. The IMMEDIATE trial reported a 0.3% HbA1c reduction at 24 weeks, [33] while Moon et al. achieved 0.8% HbA1c reduction with intermittent real-time CGM [34]. Studies with higher baseline HbA1c demonstrated larger improvements: Wright et al. (2021) found 1.5% reduction (10.1% to 8.6%) in 1,034 adults [35], while Grace and Slyer (2021) reported 3.0% reduction, both starting from ~10.1% baseline [36].

Recent meta-analyses of non-insulin-treated patients report smaller pooled HbA1c reductions of 0.31% and TIR improvements of 8.63% in non-insulin-treated patients [9], while Uhl et al. (2024) found 0.32% HbA1c reductions [37].

Our population had a low baseline HbA1c of 7.61%. This context is important for interpreting our –

0.70% reduction. Meta-regression analysis confirms that a lower baseline HbA1c (especially <7.5–8.0%) reduces apparent intervention efficacy, making expectations excessively optimistic [38]. Our −0.70% reduction is a clinically meaningful result for this specific, relatively well-controlled, non-insulin-using patients. This supports the potential for this community-based diabetes management model.

The predictive modeling analysis identified diabetes duration as the only significant independent predictor of endpoint achievement (OR 1.24 per year, 95% CI: 1.01–1.53, p = 0.040) (Table 6). Among non-achievers, those with longer diabetes duration (≥10 years) had more oral antidiabetic medications [4.00 (3.75–4.00) vs 3.00 (2.50–3.50), p = 0.07] with marginal significance, and paradoxically lower 12-week TIR [62.00% (52.50–68.00) vs 87.00% (68.50–93.45%), p = 0.025] (S3 Table). Spearman correlation confirmed a significant positive relationship between diabetes duration and number of oral medications among non-achievers (r = 0.463, p = 0.046), consistent with progressive disease complexity reducing the ability to achieve glycemic targets [39].

Notably, UKPDS 35 demonstrated that each 1% HbA1c reduction was associated with a 21% reduction in diabetes-related deaths and 37% reduction in microvascular complications [40], suggesting that the 0.70% reduction observed here, if sustained, may confer meaningful long-term clinical benefit.

Although our study design cannot distinguish between pharmacist intervention and CGM effects, the integration of both components appears to provide synergistic benefits supported by behavioral change theory. According to Bandura’s social cognitive theory, sustained behavioral change requires self-efficacy, social support, and continuous feedback [41] – elements provided through our model’s CGM data-based personalized feedback, biweekly consultations, and real-time monitoring.

Evidence supports the superiority of CGM combined with professional guidance over technology alone. Pharmacist-driven professional CGM use demonstrated greater A1c reduction when accompanied by systematic data interpretation and follow-up management [17], while CGM with virtual diabetes educator visits showed superior glycemic control (−0.69% vs −0.33% HbA1c reduction) compared to enhanced usual care [18]. In contrast, pharmacist interventions alone without CGM technology have shown variable effectiveness. While recent meta-analyses demonstrated that pharmacist-led interventions can significantly reduce HbA1c (overall standardized mean difference of −0.67, p < 0.0001) [12], studies in patients already receiving endocrinologist care found no significant HbA1c improvement when general educational interventions were provided [42].

The necessity for professional support is further highlighted by sustainability concerns. Discontinuing CGM resulted in losing approximately half of initial TIR improvements in adults with type 2 diabetes. [43] Our temporal improvement pattern (initial changes at week 3, sustained benefits through 12 weeks) suggests continuous professional guidance reinforces behavioral changes and prevents effect attenuation.

Our integrated model used CGM data to guide pharmacist counseling. This approach fits chronic care management principles, such as proactive care and patient engagement through professional support [44,45]. Implementation was aided by Korea’s National Health Insurance Service infrastructure. The PHR system allowed pharmacists to access comprehensive patient medical information (with consent). This supports the potential for broader implementation.

The model showed good patient acceptability, as evidenced by 100% participant retention. This supports its real-world feasibility. We also observed a modest trend toward fewer oral diabetes medications (−0.13, 95% CI: −0.32, 0.06, p = 0.161). This result was not statistically significant and needs more investigation in larger studies.

Stratified analysis found that treatment response was heterogeneous. Younger patients with longer diabetes duration showed the best results. We hypothesize this could be from a combination of digital health literacy and disease management experience. While younger age has been consistently associated with greater CGM adoption and engagement [46,47], our study found that diabetes duration, rather than age alone, was the only significant independent predictor of clinical reponse. This variability underscores the need for individualized patient selection. Non-achievers were more likely to be older (≥60 years, 10/19 vs 2/11), potentially reflecting lower digital engagement, more advanced β-cell dysfunction from longer disease duration [39,48], and less room for improvement due to near-target baseline HbA1c [38].

This study has several notable features. To our knowledge, this is the first study to evaluate the specific community pharmacy-led model using CGM for non-insulin-using patients with suboptimal control. This group is often underrepresented in research. Our composite endpoint design was also a strength. It addressed the methodological challenge of baseline glycemic heterogeneity. We examined both clinical and CGM metrics together, explored medication reduction trends, and identified patient predictors. Finally, the integration of Korea’s PHR system shows its potential for real-world implementation.

However, the study has several important limitations. The main limitation is the single-arm design, which had no concurrent control group. This limits causal inference about intervention effectiveness. Although we used a pre-intervention stability period, we cannot definitively attribute the improvements to our community pharmacy-led model. The results could be due to temporal factors, regression to the mean, or the Hawthorne effect from intensive monitoring [49]. Participant recruitment relied on self-referral and pharmacist outreach, raising the possibility of selection toward digitally literate and motivated individuals. Differential digital literacy may limit generalizability to older or less technology-familiar patients, who may encounter greater barriers to CGM adoption and engagement with pharmacy-led digital health services. The two participants with missing 12-week CGM data were handled using conservative imputation (mean of available weeks and LOCF), both yielding high imputed TIR values (93.9% and 100.0%). While this approach was conservative and unlikely to inflate primary findings, sensitivity analyses excluding these two participants confirmed that the primary endpoint conclusions were not materially affected. As a pilot feasibility study, our small sample size was not intended or powered to detect differences in secondary outcomes, and it constrains generalizability. We also used a convenience sampling approach. This may have introduced selection bias (e.g., more motivated participants). The 12-week intervention duration is short and provides limited data on long-term sustainability. The geographic restriction to one region in Korea also limits generalizability. Inter-pharmacist variability in counseling competency is also a potential limitation. Although all 11 pharmacists completed the same 8-session training program, individual differences in communication style, patient engagement, and diabetes management experience may have influenced patient outcomes in ways that were not formally assessed in this pilot study. Additionally, this study did not assess cost-effectiveness; future studies should evaluate the economic sustainability of this model including CGM sensor costs, pharmacist time, and digital platform integration.

It is also possible that extending the initial training phase beyond one week—particularly with greater emphasis on dietary modification and structured lifestyle counseling—could have enhanced glycemic outcomes during the biweekly follow-up period, a refinement that future trials should prospectively evaluate.

Future research priorities include randomized controlled trials with adequate sample sizes to establish efficacy compared to standard care. Longer follow-up periods are essential to assess sustainability of benefits. Cost-effectiveness analyses, investigation of optimal patient selection strategies, and standardized training protocols would support broader implementation. Large-scale implementation will require standardized training pathways, sustainable reimbursement structures, and robust digital infrastructure to support widespread CGM and PHR integration.

## Conclusions

Our pilot study showed that the community pharmacy-led model using CGM was feasible and achieved clinically meaningful glycemic improvements. The feasibility was supported by 100% participant retention. We also observed heterogeneity in treatment response across patient subgroups, warranting further investigation in adequately powered trials. The single-arm design and small sample size limit causal inference. However, these positive feasibility and preliminary effectiveness findings justify larger controlled trials to confirm efficacy and identify optimal implementation strategies.

## Supporting information

S1 File Study protocol (English). (PDF)

S2 File Study protocol (Korean). (PDF)

S3 File PLOS Human Participants Research Checklist 2025. (PDF)

S1 Table Sensitivity analysis of composite endpoint definitions. (DOCX)

S2 Table Age-stratified glycemic outcomes by primary composite endpoint achievement. (DOCX)

S3 Table Association between diabetes duration and oral medication count among non-achievers (n = 19). (DOCX)

S4 Table CONSORT 2010 checklist of information to include when reporting a pilot or feasibility trial. (DOCX)

## Abbreviations

T2DM: Type 2 Diabetes Mellitus
CGM: Continuous Glucose Monitoring
HbA1c: Glycated Hemoglobin
TIR: Time In Range
TAR: Time Above Range
TBR: Time Below Range
GMI: Glucose Management Indicator
CV: Coefficient of Variation
PHR: Personal Health Record
ADA: American Diabetes Association
EASD: European Association for the Study of Diabetes
WHO: World Health Organization
ICTRP: International Clinical Trials Registry Platform
CRIS: Clinical Research Information Service
IRB: Institutional Review Board
BMI: Body Mass Index
FBG: Fasting Blood Glucose
BP: Blood Pressure
HDL: High-Density Lipoprotein
LDL: Low-Density Lipoprotein
TG: Triglycerides
DM: Diabetes Mellitus
CI: Confidence Interval
OR: Odds Ratio
GEE: Generalized Estimating Equations
ITT: Intention-to-Treat
PP: Per-Protocol
LOCF: Last Observation Carried Forward
