# Supplementary material for: Community pharmacy-led diabetes management using continuous glucose monitoring for suboptimally controlled type 2 diabetes: A pilot feasibility study
Source: PLoS One. 2026 May 22;21(5):e0350025. doi: 10.1371/journal.pone.0350025 (PMC13196989; doi:10.1371/journal.pone.0350025)
Supplement: S1 File — (PDF) [file pone.0350025.s001.pdf]

Study Protocol: Community pharmacy-led diabetes management using continuous glucose monitoring for suboptimally controlled type 2 diabetes: A pilot feasibility study

Study Methods

① Overview of Research Methods

In this study, (1) "My Health Record (PHR) App," (2) " Health&u® 2.0 App and Administrator Web," and (3) "LibreView App and Administrator Web" programs will be used for patient counseling and management services.

The "My Health Record (PHR) App" is a platform developed and distributed by the Ministry of Health and Welfare. It provides medication history for the past year and health check-up history for the past 10 years. Counseling pharmacists plan to use this to collect preliminary patient information for counseling and management during the first face-to-face meeting, after obtaining consent for the provision of personal information from the finalized research subjects.

The " Health&u® 2.0 App and Administrator Web" is an integrated patient health management platform developed by TheJOIN Co., Ltd. It is a highly credible platform utilized in leading domestic health promotion services, such as the 'Public Health Center Mobile Healthcare' project conducted by the Korea Health Promotion Institute and the 'Wrist Doctor 9988' project in Seoul. Patients will install the Health&u® 2.0 app on their personal mobile phones and record basic lifestyle information (centered on step counts and dietary information, while other data such as sleep duration and exercise information can also be utilized). The basic lifestyle information collected through the app will be transmitted to the counseling pharmacist's administrator web via wireless communication for real-time lifestyle information monitoring.

The "LibreView App and Administrator Web" are app and web programs developed and distributed free of charge by the global pharmaceutical company Abbott. They are actively used to store and utilize measurement data from FreeStyle Libre®, a continuous glucose monitoring (CGM) system that is currently utilized as an innovative tool in the blood glucose management system for diabetic patients. This platform allows the patient's blood glucose information to be measured in real-time and transmitted to both the patient and the responsible pharmacist, enabling the monitoring of the patient's glycemic control status. Furthermore, it provides the blood glucose measurement information collected over two weeks in the form of a result report to evaluate the adequacy of blood glucose control. The result report includes information such as Time in Range (TIR), glucose statistics and targets,

glucose management indicators, 24-hour glucose profiles, and daily glucose profiles.

The utilization of all the above programs will proceed through a system where "MyData" information is delivered to the responsible pharmacist after obtaining individual consent from the patient. Additionally, high-reliability security systems such as KakaoTalk and Naver authentication systems are applied during the individual authentication process, ensuring there is no risk of personal information leakage.

FreeStyle Libre®, the CGM to be used in this study, is a medical device that can measure blood glucose without the pain of finger-pricking, unlike conventional self-monitoring of blood glucose (SMBG) methods. It can be used after attaching a sensor to a soft area with less muscle and more fat on the back of the upper arm using a simple disposable inserter. Once the sensor is attached, a small (approx. 5.5mm) filament is inserted just below the skin into the subcutaneous fat layer and fixed by a small adhesive tape, allowing it to be used for 14 days without being affected by activities such as showering. Therefore, although the sensor application process appears to involve a minor invasive procedure, FreeStyle Libre® is a product whose safety in use has been established as a medical device that individuals can directly purchase for self-glucose management through pharmacies, medical device stores, and the internet.

This study plans to involve pharmacists working in approximately 15 community pharmacies located in the Jeollabuk-do region. Each pharmacist will provide face-to-face counseling once every two weeks and non-face-to-face telephone counseling once every two weeks (totaling 6 face-to-face sessions and 6 non-face-to-face sessions) to approximately two participating patients over a total of three months. Furthermore, community pharmacists and researchers will check real-time monitoring data frequently and send individual text messages to patients as needed, providing information related to medication, proper exercise methods, and diet.

The Jeollabuk-do Pharmaceutical Association has been continuously performing academic advisory activities within the concept of community service for the development of community pharmacy pharmaceutical care services. In relation to this, an MOU agreement was signed for research projects based on community pharmacies, and research results were jointly presented in 2021. Since then, consultations and discussions for the joint execution of this research project have continued.

In the initial face-to-face counseling, it is expected to take approximately 30 minutes to 1 hour to collect and understand the patient's preliminary information. Subsequent face-to-face counseling sessions are expected to be conducted as follow-up consultations within 30 minutes, and non-face-to-face telephone consultations are expected to take within 10 minutes.

During the initial face-to-face counseling, the counseling pharmacist will have the patient install the My Health Record app, Health&u® 2.0 app, and LibreView app on their mobile phone and provide education on their usage. Additionally, the pharmacist will assist in attaching the FreeStyle Libre® sensor and educate the patient on basic precautions for the two-week usage period.

The counseling pharmacist plans to access the "MyData" of the research subjects stored in the data server through digital platforms and PDF file transmissions via messenger (KakaoTalk message function) to utilize the information during face-to-face, telephone, and text-based consultations. Access to research subjects' information is only possible for the assigned counseling pharmacist and researcher independently after obtaining the research subject's consent.

The pharmacist's counseling service will be conducted as a patient management service regarding medication, exercise, and diet. Patients will experience comprehensive disease management services including medication, exercise, and diet through professional and periodic management by the counseling pharmacist. Specifically, in medication-related services, pharmacists will provide monitoring and counseling on medication adherence management, correct administration methods, management of hypoglycemic side effects, and appropriateness reviews of newly added or changed medications during the research participation period. In the case of exercise, a daily goal for the number of steps will be set according to the patient's physical condition and age (e.g., from 7,500 to 10,000 steps per day) and managed so that the goal can be reached gradually. For diet, counseling services will be provided to ensure meals are not skipped, to guide the replacement of high-glycemic index foods with alternative foods, and to provide information and monitoring of appropriate protein intake based on body weight.

To standardize the counseling services provided by participating pharmacists to research subjects during the study process, the principal investigator will conduct preliminary training after recruiting participating pharmacists before the research begins. The preliminary training will be based on the "Latest Edition of the Clinical Practice Guidelines of the Korean Diabetes Association" and will include contents on medication guidance, exercise, and dietary counseling; the usage of (1) "My Health Record (PHR) App," (2) " Health&u® 2.0 App

and Administrator Web," and (3) "LibreView App and Administrator Web" for patient counseling and management; and protocols for preventing information leakage of research subjects. Additionally, the principal investigator plans to maintain a consistent level of pharmaceutical care services across all counseling pharmacists by monitoring the counseling services provided to patients through monthly individual interviews with participating pharmacists.

The effectiveness of the periodic counseling services provided to patients during the planned research period will be evaluated through changes in clinical laboratory values (observation periods: baseline, 3 months) and changes in individual lifestyle behaviors (observation periods: baseline, 3 months) collected before and after participation.

Clinical laboratory values include indicators such as Glycated Hemoglobin (HbA1c), Fasting Plasma Glucose, Systolic Blood Pressure, Diastolic Blood Pressure, HDL-C, LDL-C, and TG. With the consent of the research subjects, the counseling pharmacist and researcher will collect and utilize clinical test result reports confirmed by requesting tests directly from the hospitals where the research subjects are receiving treatment. The costs required for the tests will be paid through the research funds.

Additionally, values measured through the CGM will be collected as result reports every two weeks, and trends in indicators within the result reports will be compared every two weeks. In particular, the result reports generated during the first two weeks and the last two weeks will be compared as major indicators to evaluate the adequacy and optimization of blood glucose management. The measured values are as follows: Time in Range (TIR); glucose statistics and targets (average glucose during the wearing period, glucose range and target values, glucose variability, and Glucose Management Indicator (GMI)); glucose management indicators (estimated A1c based on average glucose levels from measurements over the last 14 days); 24-hour glucose profile (identifying glucose fluctuation ranges by finding patterns of hyperglycemia and hypoglycemia); and daily glucose profile (a method for identifying specific daily glucose patterns to help identify the causes of glucose deviations).

Used CGM sensors will be collected and disposed of by the counseling pharmacist during the face-to-face counseling visits.

The measurement of the degree of change in individual lifestyle behavior will be evaluated

through questionnaire forms regarding knowledge of diabetes and self-management (diabetes management knowledge, medication-related knowledge), behavior (proficiency in self-monitoring and management of blood glucose), self-management ability index (medication adherence, self-monitoring of blood glucose, diet, exercise, weight control, hypoglycemia management), and satisfaction with pharmacist services and smart device usage. These questionnaires will be distributed and evaluated by the counseling pharmacist during face-to-face meetings at the start and end of the study.

The composition of the questionnaire for research subjects was based on questionnaires from previous studies and reconstructed after discussion with co-investigators.

During the research process, participants can voluntarily decide to stop participating at any time if they wish, and there will be no disadvantages for doing so. Additionally, upon withdrawal of participation, all personal information collected up to that point will be destroyed.

Research subjects will be provided with a CGM device (market value approximately 100,000 KRW) free of charge for each of the 6 face-to-face visits. Additionally, a total of 20,000 KRW will be paid for blood test costs (pre and post-participation, twice), and upon final completion of research participation, a patient gift (a gift certificate or cash worth 30,000 KRW) in the nature of transportation expenses will be provided. If participation is discontinued midway, the post-participation blood test costs and the patient gift will not be provided separately.

② Recruitment

Method for Research Subjects Research subjects who meet the inclusion criteria will be recruited through the Jeollabuk-do Pharmaceutical Association, up to a maximum of 30 individuals.

③ Inclusion and Exclusion Criteria for Research Subjects

Inclusion Criteria: Patients with Type 2 Diabetes aged between 45 and 65 who have confirmed HbA1c levels within 12 months prior to the start of the study, have a history of taking two or more types of diabetes medications within the last year, yet have HbA1c levels of 6.5% or higher, and who use a smartphone.

Exclusion Criteria: Patients who have taken fewer than two types of diabetes medications within the last year; patients with Type 1 Diabetes; patients currently undergoing insulin therapy; patients who meet the inclusion criteria but have difficulty participating due to a lack of proficiency in using smartphones or applications; patients who cannot walk or have difficulty exercising; patients who do not use a smartphone. Patients for whom continuous participation is difficult due to skin damage such as allergic reactions or other side effects resulting from the attachment of the CGM device.

④ Target Number of Research Subjects and Rationale for Calculation

This study intends to recruit and proceed with a maximum of 30 patients with Type 2 Diabetes, referring to the scale of research funds and results of recent preliminary research surveys. Calculated based on Cohen's Power analysis using the G*Power 3.1 program (t-test, matched pairs analysis, significance level , effect size , power), the minimum number of research subjects is 27. Considering a dropout rate of approximately 10%, a scale of 30 is considered appropriate.

⑤ Procedure for Obtaining Consent from Research Subjects

Research subjects will voluntarily apply for the pharmacist's patient management service and provide their diabetes-related clinical information after completing the consent procedure for the provision of personal information during the first face-to-face consultation with the pharmacist. During the consent process, the researcher plans to explain how the patient's clinical information will be utilized and stored, as well as the disposal method after the completion of the study. Additionally, it will be explained that research subjects can withdraw their participation and that all previously collected personal information data will be destroyed upon withdrawal.

⑥ Principles and Methods of Statistical Analysis

Statistical analysis of the research results will be conducted using IBM SPSS Statistics ver. 23.0 (IBM Corp. Armonk, NY, USA).

Analysis Methods

- Descriptive Statistics: For basic demographic variables such as gender and age, clinical laboratory values such as blood pressure and blood cholesterol levels, and glucose indicators such as TIR measured from the CGM at the start of the study, 2-

week intervals, and the end of the study, continuous variables will be calculated as mean and standard deviation, and nominal variables as frequency and percentage (%).

- Effect Size Analysis: Differences before and after the intervention will be quantitatively evaluated, and statistical significance will be determined when the p-value is less than 0.05.
- Paired t-test:
  - *Purpose:* To test the difference in means before and after the intervention.
  - *Analysis Method:* Compare the differences before and after the intervention in blood glucose data measured through CGM, HbA1c levels, and fasting plasma glucose.

- Repeated Measures ANOVA:
  - *Purpose:* To evaluate differences in glucose indicator measurements repeated across multiple time points during the intervention period.
  - *Analysis Method:* Perform a test of mean differences using the F-statistic.

- Multivariate Regression Analysis:
  - *Purpose:* To identify independent variables that predict the effect of the intervention.
  - *Analysis Method:* Analyze factors affecting nominal outcome variables (e.g., improvement in TIR) using logistic regression analysis considering multiple variables simultaneously, and evaluate the impact of the intervention on continuous outcome variables (blood glucose levels, reduction in HbA1c) using multiple linear regression analysis.

- Time-Series Analysis:
  - *Purpose:* To analyze patterns of change over time in glucose indicators collected at 2-week intervals.
  - *Analysis Method:* Apply basic time-series models using the moving average method and Auto-Regressive Integrated Moving Average (ARIMA) models, and conduct the Dickey-Fuller test.

⑦ Observation Items and Observation/Test Methods

- Personal information voluntarily provided by the research subject (name, phone number) and clinical laboratory values (age, gender, underlying diseases, height, weight, duration of diabetes, type of diabetes medication, insulin administration status, other medications, alcohol consumption, smoking status, presence of hypertension, presence of hyperlipidemia, body mass index, list of medications, self-monitored blood glucose levels, fasting plasma glucose, HbA1c, systolic blood pressure, diastolic blood pressure, HDL-C, LDL-C, TG, dietary information, step counts, activity (exercise) time, etc.).

- Data collected through the CGM: Time in Range (TIR), glucose statistics and targets (average glucose during the wearing period, glucose range and target values, glucose variability, Glucose Management Indicator (GMI)), glucose management indicators (estimated HbA1c based on average glucose levels from the last 14 days), 24-hour glucose profile (identifying glucose fluctuation ranges by finding patterns of hyperglycemia and hypoglycemia), and daily glucose profile (identifying specific daily glucose patterns to help determine the cause of glucose deviations).

- Research subjects' knowledge and awareness regarding diabetes and self-management (diabetes-related knowledge, medication-related knowledge, exercise and dietary management awareness, etc.), self-management ability index and behavior (medication adherence, diet, exercise, weight control, hypoglycemia management, proficiency in blood glucose measurement and management, etc.), and satisfaction with pharmacist services and smart device usage.

⑧ Evaluation and Interpretation Methods

After the pharmacist's patient management service is provided to the research subjects during the research period, the clinical effectiveness of the patient management service conducted by community pharmacists will be evaluated by comparing the pre-test and post-test values of clinical laboratory results and individual lifestyle behavior information.

⑨ Reporting Method

The final research results will be reported through presentations at academic conferences and

publication in journals.
