# Supplementary material for: Community pharmacy-led diabetes management using continuous glucose monitoring for suboptimally controlled type 2 diabetes: A pilot feasibility study
Source: PLoS One. 2026 May 22;21(5):e0350025. doi: 10.1371/journal.pone.0350025 (PMC13196989; doi:10.1371/journal.pone.0350025)
Supplement: S2 File — (PDF) [file pone.0350025.s002.pdf]

Study Protocol: Community pharmacy-led diabetes management using continuous glucose monitoring for suboptimally controlled type 2 diabetes: A pilot feasibility study

연구방법

① 연구방법 개요

• 본 연구에서는 환자 상담 및 관리 서비스를 위해 (1) "나의건강기록(PHR) 앱"과 (2) "헬스앤유2.0 앱 및 관리자웹", (3) "리브레뷰 앱 및 관리자웹" 프로그램을 사용할 예정이다.

• 나의건강기록(PHR) 앱은 보건복지부에서 개발하여 배포한 플랫폼으로 환자의 최근 1년간 투약정보 이력 및 최근 10년 이내의 건강검진 이력 정보를 제공하며, 상담약사는 연구대상자로 최종 선정된 연구대상자에 대해 개인정보제공 동의를 거친 후 이를 확인함으로써 환자와의 첫 대면 시 상담 및 관리를 위한 환자의 사전 정보 수집에 이용할 계획이다.

• 헬스앤유2.0앱 및 관리자웹은 ㈜ 더조인에서 개발한 통합형 환자 건강관리 플랫폼으로 한국건강증진개발원에서 진행 중인 '보건소 모바일헬스케어' 사업 및 서울특별시에 진행 중인 '손목닥터9988'과 같은 국내 대표적인 건강증진서비스에 활용되는 공신력 있는 플랫폼이다. 환자는 개인 핸드폰에 헬스앤유2.0앱을 설치한 후 환자의 기본 생활정보(걸음수 및 식이정보 중심, 기타 수면시간 및 운동정보 등 활용 가능)를 기록할 예정이며, 앱을 통해 수집된 기본 생활정보는 핸드폰 무선통신을 통해 상당약사의 관리자웹으로 전송되어 실시간 생활정보 모니터링을 진행할 계획이다.

• 리브레뷰 앱 및 관리자웹은 글로벌 제약사인 애보트(Abbott)에서 개발하여 무료로 배포하고 있는 앱과 웹 프로그램으로 최근 당뇨병 환자의 혈당관리 체계에서 혁신적인 도구로 활용되고 있는 연속혈당측정기인 프리스타일 리브레®의 측정데이터를 보관 및 활용하기 위해 활발히 사용되고 있다. 이 플랫폼은 환자의 혈당 정보를 실시간 측정하여 환자 본인 및 담당 약사에게도 전송하여 환자의 혈당 조절 상태를 모니터링 할 수 있게 한다. 또한, 2주 간 수집된 혈당 측정정보를 결과보고서 형태로 제공하여 혈당 조절의 적절

성을 평가할 수 있도록 한다. 결과보고서는 목표 혈당 범위내로 유지된 시간 비율, 혈당 통계 및 목표값, 혈당 관리 지표, 24시간 혈당 프로파일, 일일 혈당 프로파일 정보 등이 제시된다.

• 위의 모든 프로그램의 활용은 환자의 개별 동의를 거친 후 MyData 정보를 담당 약사에게 전달하는 체계로 진행될 계획이다. 또한, 개별 인증 과정에서 카카오톡 및 네이버 인증 체계 등 신뢰도 높은 보완 체계가 적용되어 개인정보 유출에 대한 위험성은 없다.

• 본 연구에서 사용할 연속혈당측정기인 프리스타일 리브레®는 기존의 자가혈당측정방법과는 달리 손가락 채혈의 고통이 없이 혈당을 측정할 수 있는 의료기기로 간단한 일회용 삽입 장치를 사용하여 팔 위쪽(상박)의 뒷부분에 근육이 적고 살이 많은 말랑한 부위에 센서를 부착한 후 사용할 수 있다. 센서를 부착하게 되면, 작은(약 5.5mm) 필라멘트가 피부 바로 아래 피하지방층에 삽입되고, 작은 접착테이프에 의해 고정되어 14일간 샤워 등에 영향을 받지 않고 사용하게 된다. 따라서 센서 착용 과정에서 미세한 침습 과정이 되는 것으로 보이나, 프리스타일 리브레®는 현재 약국 및 의료기기 판매점, 인터넷 등을 통해 개인이 직접 자가 혈당관리 목적으로 구매할 수 있는 의료기기로 사용상의 안전성이 확보된 제품이다.

• 본 연구는 전라북도 지역에 위치한 15개 내외의 지역약국에 근무하는 약사가 2명 내외의 참여환자에게 총 3개월에 걸쳐서 2주 1회 대면 상담, 2주 1회 비대면 전화상담(대면 상담 6회와 비대면 상담 6회)을 제공하는 형태로 진행할 계획이다. 또한, 지역약국 약사와 연구자는 실시간 모니터링 자료를 수시로 확인한 후 약물 복약 관련 정보 및 올바른 운동법, 식이에 관한 문자메시지를 필요에 따라 개별적으로 환자에게 전송할 계획이다.

• 전라북도약사회는 본 연구자가 지역약국 약료서비스 발전을 위해 지역사회 봉사 개념 가운데 지속적으로 학술관련 자문활동을 수행해 오고 있다. 관련하여 지역약국 기반 연구사업을 위한 MOU 협약을 맺고 2021년 연구결과를 함께 발표한 바 있으며, 이후에도 본 연구사업의 공동 진행을 위한 협의와 논의를 지속해 왔다.

• 초회 대면상담에서는 환자의 사전 정보 수집 및 파악을 위해 30분~1시간 가량의 상담 시간이 소요될 것으로 예상된다. 이후 진행되는 대면 상담에서는 30분 이내의 follow-up 형태의 상담이 진행될 것으로 예상되며, 비대면 전화상담에서는 10분 이내의 상담이 진행될 것으로 예상된다.

• 초회 대면 상담에서 상담 약사는 환자의 핸드폰에 나의건강기록 앱, 헬스앤유2.0 앱, 리브레뷰 앱을 설치하도록 한 후 사용법에 대해 교육할 것이다. 그리고 프리스타일 리브레® 센서를 부착하도록 하며 2주 간의 사용 기간 동안 기본적인 주의사항에 대해 교육할 것이다.

• 상담 약사는 데이터 서버에 저장된 연구대상자의 MyData를 디지털 플랫폼 및 PDF 파일의 메신저 전송(카카오톡 메시지 기능) 등의 방법을 통해 조회하여 환자 대면 시와 유선 상담 시, 필요에 따라 문자 메시지를 통한 상담 시에 활용하여 상담을 진행할 계획이다. 연구대상자의 정보 조회는 연구대상자의 동의 후 환자에게 배정된 상담 약사와 연구자만 독립적으로 조회가 가능하다.

• 약사의 상담서비스는 복약 및 운동, 식이에 관한 환자관리 서비스로 진행할 계획이다. 환자는 상담 약사의 전문적이고 주기적인 관리를 통해 복약 및 운동, 식이 등 전반적인 질환관리 서비스를 경험하게 된다. 특히, 상담 약사는 복약관련 서비스에서 복약순응도 관리와 정확한 복용법 관리, 저혈당 부작용 발생, 연구 참여 기간 중 신규 추가 혹은 변경 약물에 대한 적절성 검토 등에 모니터링 및 상담서비스를 제공하게 된다. 운동의 경우 일일 목표 걸음수를 환자의 신체 및 연령에 따라 목표(예, 1일 7,500보에서 10,000보)를 설정하여 점차적으로 목표에 도달할 수 있도록 관리하게 된다. 식이의 경우 식사를 거르지 않도록 하는 것과 당지수가 높은 음식을 대체음식으로 변경하도록 안내하는 것, 체중에 따른 적절한 단백질 섭취량에 대한 정보제공 및 섭취 모니터링 등의 상담서비스를 제공할 예정이다.

• 연구책임자는 연구진행 과정에서 참여약사의 연구대상자에 대한 상담서비스를 균일화

하기 위해 연구 진행 전 참여약사를 모집한 이후 사전교육을 시행할 예정이다. 사전교육은 ‘대한당뇨병학회 진료지침 가이드라인 최신판’에 근거하여 복약지도 및 운동, 식이상담 등에 관한 내용, 환자 상담 및 관리 서비스를 위해 (1) “나의건강기록(PHR) 앱”과 (2) “헬스앤유2.0 앱 및 관리자웹”, (3) “리브레뷰 앱 및 관리자웹” 프로그램의 사용법에 대한 내용, 연구대상자에 대한 정보유출 방지에 대한 내용 등으로 구성하여 진행할 예정이다. 또한, 연구책임자는 매월 참여약사와의 개별 면담을 통해 환자와 진행 중인 상담서비스 내용에 대해 모니터링 함으로써 모든 상담 약사들의 약료서비스 수준을 일관성 있게 유지할 계획이다.

• 계획된 연구기간 동안 환자에게 제공된 주기적 상담서비스의 효과는 연구대상자의 연구 참여 전과 연구 종료 후에 수집된 임상검사 수치(관찰시기: base-line, 3month) 및 개인 생활 행태에 대한 변화 정도 측정(관찰시기: base-line, 3month)을 통해 평가할 계획이다.

• 임상검사 수치는 당화혈색소(HbA1c), 공복혈당, 수축기 혈압, 이완기 혈압, HDL-C, LDL-C, TG 등의 검사지표에 대해 연구대상자가 진료 받고 있는 병원에 직접 검사를 의뢰하여 확인된 임상검사 결과지를 연구대상자의 동의를 통해 상담 약사와 연구자가 수집하여 활용할 예정이며, 검사에 필요한 검사비용은 연구비를 통해 지급될 예정이다.

• 또한, 연속혈당측정기를 통해 측정된 값들은 2주 마다 결과지를 수집할 계획이며, 결과지의 지표들의 변화 양상을 2주 간격으로 비교할 계획이며, 특히 첫 2주와 마지막 2주 동안 생성된 결과지를 주요 지표로 비교하여 혈당관리의 적절성 및 최적화 여부를 평가할 계획이다. 측정되는 값들은 다음과 같다. 목표 혈당 범위내로 유지된 시간 비율(Time in Range, TIR), 혈당 통계 및 목표값(착용한 기간 동안의 평균 혈당, 혈당 범위 및 목표값, 혈당 변동성, 혈당 관리 표시기(GMI)), 혈당 관리 지표(최근 14일 동안의 측정값에서 평균 혈당 수치에 기반한 환자의 추정 A1c), 24시간 혈당 프로파일(고혈당과 저혈당의 패턴을 찾아서 혈당 변동폭을 확인함), 일일 혈당 프로파일(구체적인 일일 혈당 패턴을 확인하는 방법으로서, 혈당 이탈의 원인을 규명할 수 있도록 도움).

• 사용이 완료된 연속혈당측정기의 경우 대면상담을 위한 방문 시 상담약사가 수거하여

폐기할 계획이다.

• 연구대상자의 개인 생활 행태에 대한 변화 정도 측정은 당뇨병 질환 및 자가관리에 대한 지식도(당뇨병 관리 지식, 복용 약물 관련 지식), 행위도(자가혈당 측정 및 관리 숙련도), 자가관리능력 지수(복약순응도, 자가혈당 측정, 식사, 운동, 체중조절, 저혈당 관리), 약사 서비스 및 스마트 기기 사용에 대한 만족도에 관한 설문지 양식을 연구 시작 전과 연구 종료 시점에 상담약사가 환자와의 대면 시 배부하여 평가할 계획이다.

• 연구대상자에 대한 설문지 구성은 선행연구의 설문지를 참조하였으며, 공동연구자와 논의 후 재구성하였다.

• 연구진행 과정에서 연구 참여자가 원하는 경우 언제든지 자발적인 결정에 따라 참여 중단이 가능하며, 참여 중단 시에도 어떠한 불이익이 없다. 또한 참여 철회 시 그동안 수집된 개인정보는 모두 폐기할 예정이다.

▪ 연구대상자는 6회의 대면 방문에 대해 매회 시중가 10만원 상당의 연속혈당측정기기를 무상으로 지급할 예정이다. 또한, 혈액검사비용으로 사전, 사후 2회에 대해 총 2만원을 지급하며, 연구 참여를 최종적으로 완수할 경우 교통연비 포함 성격의 환자 답례품(3만원 상당의 상품권이나 현금)을 지급할 예정이다. 연구참여를 중도에 포기할 경우에는 사후 혈액검사비용과 환자 답례품을 별도로 지급하지 않을 예정이다.

② 연구대상자 모집 방법

▪ 선정기준에 해당하는 연구대상자는 전라북도약사회를 통해 최대 30명으로 모집할 예정이다.

③ 연구대상자의 선정기준, 제외기준

▪ **연구대상자 선정기준**: 연구 개시 전 12개월 이내의 당화혈색소 수치를 확인할 수 있고, 최근 1년 간 2종 이상의 당뇨약을 복용한 이력이 있음에도 당화혈색소 수치가 6.5% 이상인 45세 이상 65세 미만의 2형당뇨병 환자로 스마트폰을 사용하는 환자.

▪ **연구대상자 제외기준**: 최근 1년간 2종 미만의 당뇨약을 복용한 환자, 1형당뇨병 환자, 인슐린을 투여 중인 환자, 선정기준에 해당하나 스마트폰이나 어플의 원활한 사용 능력이 떨어져 참여가 곤란한 환자, 걸음을 걸을 수 없고 운동이 어려운 환자, 스마트폰을 사용하지 않는 환자. 연속혈당측정기기의 부착에 따른 피부 알레르기 반응 등의 손상이나 기타 부작용이 발생하여 지속적인 연구참여가 어려운 환자.

④ 목표 연구대상자의 수 및 산출근거

▪ 본 연구는 연구비 규모와 최근 진행한 사전 연구내용 조사결과들을 참조하여 최대 30명의 2형당뇨병 환자를 모집하여 진행하고자 한다. Cohen의 Power analysis를 근거로 G*Power3.1 프로그램을 이용하여 산출한 결과(t-test, matched pairs 분석 시 유의수준 α=0.05, 효과크기 0.5, 검정력 0.7) 최소 연구대상자가 27명으로 중도탈락자를 10% 정도로 감안할 때 적절한 규모로 사료 된다.

⑤ 연구대상자의 동의 취득 절차

▪ 연구대상자는 자발적으로 약사의 환자관리 서비스를 신청하고 약사와의 첫 대면 상담 시 본인 정보 제공 동의 절차를 마친 후 대상자 본인의 당뇨병 관련 임상정보를 제공하게 된다. 연구자는 본인 정보 제공 동의 절차를 진행하는 과정에서 환자의 임상정보 활용 방법과 보관방법, 연구 종료 후의 폐기 방법에 대해 설명할 계획이다. 또한, 연구대상자가 연구참여를 철회할 수 있음을 설명하고, 참여 철회 시 기존에 수집된 모든 개인정보 자료도 함께 폐기됨을 설명할 계획이다.

⑥ 통계분석 원칙 및 방법

▪ 연구결과는 IBM SPSS Statistics ver. 23.0 (IBM Corp. Armonk, NY, USA)을 이용하여 통계 분석을 시행할 예정이다.

▪ 분석 방법

1. 기술 통계

성별, 나이 등 기본 인구학적 변수, 혈압, 혈중 콜레스테롤 수치 등 임상검사 수치, 연속혈당측정기기로부터 측정된 연구 개시 시점, 2주 간격 및 최종 연구종료 시점에서의 TIR 등의 혈당 지표에 대해 연속형 변수는 평균, 표준편차, 명목형 변수는 빈도 및 백분율(%) 계산하고자 한다.

2. 효과 크기 분석: 중재 전후의 차이를 정량적으로 평가하고자 하며, 통계적 유의성은 P-value 값이 0.05 미만일 때 유의한 것으로 판단하고자 한다.

가. Paired t-test

1) 목적: 중재 전후의 평균 차 검정

2) 분석 방법: 연속혈당측정기기를 통해 측정된 혈당 데이터, 당화혈속 수치 및 공복혈당 등에서 중재 전후 차이를 비교.

나. 반복측정 ANOVA

1) 목적: 중재 기간 동안 여러 시점에 걸쳐 반복된 혈당지표 측정치의 차이 평가

2) 분석 방법: F 통계량을 이용한 평균차 검정을 시행

다. 다변량 회귀분석

1) 목적: 중재 효과를 예측하는 독립변수 식별

2) 분석 방법: 여러 변인을 동시에 고려한 로지스틱 회귀분석을 이용하여 명목형 결과 변수(TIR의 개선 여부 등)에 영향을 미치는 요인 분석 및 다중 선형 회구분석을 사용하여 연속형 결과 변수(혈당 수치, 당화혈색소 감소량)에 대한 중재의 영향 평가

라. 시계열 분석

1) 목적: 2주 간격으로 수집된 혈당 지표의 시간에 따른 변화의 패턴 분석

2) 분석 방법: 이동 평균법을 이용한 기초적 시계열 모델 및 자기회귀 누적 이동평균(ARIMA) 모델을 적용하고 Dickey-Fuller 검정

⑦ 관찰항목, 관찰 검사 방법

▪ 연구대상자가 자발적으로 제공한 개인정보(이름, 전화번호) 및 임상검사수치(나이, 성별, 기저질환, 키, 체중, 당뇨병 유병기간, 당뇨약 종류, 인슐린 투여 여부, 기타 복용약물, 음주 여부, 흡연 여부, 고혈압 여부, 고지혈증 여부, 체질량 지수, 복용 약물 목록, 자가혈당측정 수치, 공복혈당, 당화혈색소, 수축기 혈압, 이완기 혈압, HDL-C, LDL-C, TG, 식이 상태 정보, 걸음수, 활동(운동)시간 등)

▪ 연속혈당측정기기를 통해 수집된 데이터: 목표 혈당 범위내로 유지된 시간 비율(Time in Range, TIR), 혈당 통계 및 목표값(착용한 기간 동안의 평균 혈당, 혈당 범위 및 목표값, 혈당 변동성, 혈당 관리 표시기(GMI)), 혈당 관리 지표(최근 14일 동안의 측정값에서 평균 혈당 수치에 기반한 환자의 추정 HbA1c), 24시간 혈당 프로파일(고혈당과 저혈당의 패턴을 찾아서 혈당 변동폭을 확인함), 일일 혈당 프로파일(구체적인 일일 혈당 패턴을 확인하는 방법으로서, 혈당 이탈의 원인을 규명할 수 있도록 도움)

▪ 연구대상자의 당뇨병 질환 및 자가관리에 대한 지식도 및 인지도(당뇨병 관련 지식, 복용 약물 관련 지식, 운동 및 식이 관리 인식도 등), 자가관리능력 지수 및 행위도(복약순응도, 식사, 운동, 체중조절, 저혈당 관리, 혈당 측정 및 관리 숙련도 등), 약사 서비스 및 스마트 기기 사용에 대한 만족도

⑧ 평가 방법 및 해석 방법

▪ 연구기간 동안 연구대상자에게 약사의 환자관리 서비스가 진행된 후 임상검사 수치 및 개인 생활 행태 정보에 대한 사전 검사 결과값과 사후 결과값을 상호 비교하여 지역약국

약사가 진행한 환자관리 서비스의 임상적 효과를 평가하고자 한다.

⑨ 보고 방법

▪ 최종 연구결과에 대해 학술대회 발표 및 논문 발표를 통해 보고할 예정이다.
