# Supplementary material for: Community pharmacy-led diabetes management using continuous glucose monitoring for suboptimally controlled type 2 diabetes: A pilot feasibility study
Source: PLoS One. 2026 May 22;21(5):e0350025. doi: 10.1371/journal.pone.0350025 (PMC13196989; doi:10.1371/journal.pone.0350025)
Supplement: S1 Table — (DOCX) [file pone.0350025.s004.docx]

# Supplementary Material

### Supplementary Table S1. Sensitivity Analysis of Composite Endpoint Definitions

| **Endpoint Definition** | **N** | **Achievers** | **Rate (%)** | **95% CI** |
| --- | --- | --- | --- | --- |
| TIR threshold: >65% | 30 | 12 | 40.00 | 24.58–57.71 |
| TIR threshold: >75% | 30 | 10 | 33.33 | 19.16–51.19 |
| HbA1c: absolute reduction only | 30 | 11 | 36.67 | 21.87–54.49 |
| HbA1c: relative reduction only | 30 | 9 | 30.00 | 16.69–47.93 |
| TIR: 4-week average (weeks 9-12) | 30 | 11 | 36.67 | 21.87–54.49 |
| All endpoint definitions are variants of the primary composite endpoint, with one component modified as specified: Primary composite endpoint: HbA1c ≤7.0% AND meaningful HbA1c reduction (≥0.5% absolute OR ≥10% relative) AND TIR >70% at week 12.  Endpoint variants: TIR threshold variants (rows 1-3): Modified TIR criterion (>65%, >70%, or >75%) while maintaining other components unchanged; HbA1c reduction variants (rows 4-5): Modified HbA1c improvement criterion (absolute only or relative only) while maintaining TIR >70% and HbA1c ≤7.0%; TIR measurement variant (row 6): TIR calculated as mean of weeks 9-12 instead of week 12 only.  Data presented as number (percentage) with 95% confidence intervals using Wilson method.  TIR = time in range (70-180 mg/dL). | | | | |
