# Supplementary material for: Community pharmacy-led diabetes management using continuous glucose monitoring for suboptimally controlled type 2 diabetes: A pilot feasibility study
Source: PLoS One. 2026 May 22;21(5):e0350025. doi: 10.1371/journal.pone.0350025 (PMC13196989; doi:10.1371/journal.pone.0350025)
Supplement: S2 Table — (DOCX) [file pone.0350025.s005.docx]

# Supplementary Material

### Supplementary Table S2. Age-Stratified Glycemic Outcomes by Primary Composite Endpoint Achievement

| **Variable** | **Age <60 years (N=18)** | | **Age ≥60 years (N=12)** | |
| --- | --- | --- | --- | --- |
| **Variable** | **Achievers (n=9)** | **Non-Achievers (n=9)** | **Achievers (n=2)** | **Non-Achievers (n=10)** |
| **Primary Endpoint Achievement** | | | | |
| Achievement rate, n (%) | 9/18 (50.0%) | | 2/12 (16.7%) | |
| 95% CI (Clopper-Pearson) | (26.0–74.0%) | | (2.1–48.4%) | |
| Between-group comparison (Fisher's exact) | p = 0.121 (age <60 vs ≥60) | | | |
| **Glycemic Outcomes at 12 Weeks** | | | | |
| HbA1c change, % | −1.30 (−2.00 to −1.00) | −0.20 (−0.50 to 0.00) | −0.75 (−0.83 to −0.68) | −0.25 (−0.40 to −0.05) |
| p-value (within stratum) | <0.001 | | — | |
| TIR change, %pᵃ | 17.00 (11.00 to 19.00) | −1.00 (−4.00 to 7.00) | 13.00 (5.50 to 20.50) | 5.00 (1.66 to 6.00) |
| p-value (within stratum) | 0.008 | | — | |
| TAR change, %p | −17.00 (−27.00 to −13.00) | 0.00 (−6.00 to 4.00) | −12.50 (−20.25 to −4.75) | −4.00 (−6.75 to −1.50) |
| p-value (within stratum) | 0.009 | | — | |
| Data are presented as median (Q1–Q3) for continuous variables. Between-group comparisons (Achievers vs Non-Achievers within each age stratum) by Mann-Whitney U test.  — : Statistical comparison not performed for the ≥60 years stratum due to small Achiever sample size (n=2).  ᵃ TIR outcomes: one Achiever (id=21) in the <60 years group had missing 12-week CGM data and was imputed using LOCF (last observed TIR carried forward).  Abbreviations: CGM, continuous glucose monitoring; HbA1c, glycated hemoglobin; LOCF, last observation carried forward; TAR, time above range; TIR, time in range. | | | | |
