# Supplementary material for: Community pharmacy-led diabetes management using continuous glucose monitoring for suboptimally controlled type 2 diabetes: A pilot feasibility study
Source: PLoS One. 2026 May 22;21(5):e0350025. doi: 10.1371/journal.pone.0350025 (PMC13196989; doi:10.1371/journal.pone.0350025)
Supplement: S3 Table — (DOCX) [file pone.0350025.s006.docx]

# Supplementary Material

### Supplementary Table S3. Association Between Diabetes Duration and Oral Medication Count Among Non-Achievers (n=19)

| **Variable** | **DM duration ≥10 years (n=8)** | **DM duration <10 years (n=11)** | **p-value** |
| --- | --- | --- | --- |
| **Patient Characteristics** | | | |
| DM duration, years | 15.00 (13.00–17.00) | 5.00 (3.50–6.50) | — |
| Age, years | 61.00 (58.50–62.25) | 58.00 (52.00–61.00) | 0.184 |
| Baseline HbA1c, % | 7.35 (6.88–8.68) | 6.90 (6.75–7.50) | 0.441 |
| No. of oral DM medications | 4.00 (3.75–4.00) | 3.00 (2.50–3.50) | 0.070 |
| **Glycemic Outcomes at 12 Weeks** | | | |
| HbA1c change, % | −0.20 (−0.62–0.00) | −0.30 (−0.45–0.10) | 0.781 |
| 12-week TIR, %ᵃ | 62.00 (52.50–68.00) | 87.00 (68.50–93.45) | 0.025 |
| TIR change, %p | 1.00 (−10.75–6.75) | 4.00 (−0.06–6.50) | 0.631 |
| **Correlation Analysis (All Non-Achievers, n=19)** | | | |
| **Spearman r (DM duration × No. of oral DM medications)** | r = 0.463, p = 0.046 | | |
| Data are presented as median (Q1–Q3). Between-group comparisons by Mann-Whitney U test.  — : No between-group comparison applicable (DM duration is the grouping variable).  All analyses are exploratory. Results should be interpreted with caution given the small sample size (n=19).  ᵃ 12-week TIR: one participant (id=20, <10yr group) had missing CGM data; imputed using mean of weeks 11 and 12.  Abbreviations: BMI, body mass index; CGM, continuous glucose monitoring; DM, diabetes mellitus; HbA1c, glycated hemoglobin; TAR, time above range; TIR, time in range. | | | |
